# Functional interrogation of *Plasmodium* genus metabolism identifies species- and stage-specific differences in nutrient essentiality and drug targeting

**DOI:** 10.1371/journal.pcbi.1005895

**Published:** 2018-01-04

**Authors:** Alyaa M. Abdel-Haleem, Hooman Hefzi, Katsuhiko Mineta, Xin Gao, Takashi Gojobori, Bernhard O. Palsson, Nathan E. Lewis, Neema Jamshidi

**Affiliations:** 1 King Abdullah University of Science and Technology (KAUST), Computational Bioscience Research Centre (CBRC), Thuwal, Saudi Arabia; 2 King Abdullah University of Science and Technology (KAUST), Biological and Environmental Sciences and Engineering (BESE) division, Thuwal, Saudi Arabia; 3 Department of Bioengineering, University of California, San Diego, La Jolla, CA, United States of America; 4 Novo Nordisk Foundation Center for Biosustainability at the University of California, San Diego School of Medicine, La Jolla, CA, United States of America; 5 Department of Pediatrics, University of California, San Diego, La Jolla, CA, United States of America; 6 Institute of Engineering in Medicine, University of California, San Diego, La Jolla, CA, United States of America; 7 Department of Radiological Sciences, University of California, Los Angeles, CA, United States of America; Chalmers University of Technology, SWEDEN

## Abstract

Several antimalarial drugs exist, but differences between life cycle stages among malaria species pose challenges for developing more effective therapies. To understand the diversity among stages and species, we reconstructed genome-scale metabolic models (GeMMs) of metabolism for five life cycle stages and five species of *Plasmodium* spanning the blood, transmission, and mosquito stages. The stage-specific models of *Plasmodium falciparum* uncovered stage-dependent changes in central carbon metabolism and predicted potential targets that could affect several life cycle stages. The species-specific models further highlight differences between experimental animal models and the human-infecting species. Comparisons between human- and rodent-infecting species revealed differences in thiamine (vitamin B1), choline, and pantothenate (vitamin B5) metabolism. Thus, we show that genome-scale analysis of multiple stages and species of *Plasmodium* can prioritize potential drug targets that could be both anti-malarials and transmission blocking agents, in addition to guiding translation from non-human experimental disease models.

## Author summary

Malaria kills nearly one-half million people a year and over 1 billion people are at risk of becoming infected by the parasite. Plasmodial infections are difficult to treat for a myriad of reasons, but the ability of the organism to remain latent in hosts and the complex life cycles greatly contributed to the difficulty in treat malaria. Genome-scale metabolic models (GeMMs) enable hierarchical integration of disparate data types into a framework amenable to computational simulations enabling deeper mechanistic insights from high-throughput data measurements. In this study, GeMMs of multiple Plasmodium species are used to study metabolic similarities and differences across the Plasmodium genus. *In silico* gene-knock out simulations across species and stages uncovered functional metabolic differences between human- and rodent-infecting species as well as across the parasite’s life-cycle stages. These findings may help identify drug regimens that are more effective in targeting human-infecting species across multiple stages of the organism.

## Introduction

Malaria is a worldwide problem of clinical significance causing an estimated 483,000 deaths, with a disproportionate percentage occurring in children less than 5 years of age, according to the World Health Organization[1]. Additionally, 1.2 billion people are at high risk of contracting the infection[1]. Plasmodium is a challenging organism to understand and treat, since it has a complex life cycle[2] and can remain latent within hosts. Indeed, current antimalarials target the symptomatic Plasmodium life cycle stages, while, allowing ample time for transmission before symptoms are seen. The use of experimental model organisms, such as mice, has provided a wealth of knowledge about the various life cycle stage in the Plasmodium genus; however many differences between rodent-, primate-, and human-infecting species remain incompletely understood. Thus, to identify effective means to eradicate malaria, there is a need to understand its biological capabilities as it relates to drug targeting in different stages of its life cycle and also across the different species.

Among potential drug targets, metabolic genes are of particular interest, since many anabolic and catabolic processes are critical for cellular growth and survival. Furthermore, methods have been developed to identify vulnerabilities in human pathogens by accurately predicting essential metabolic genes in genome-scale metabolic network reconstructions [3–14]. Here, we present detailed genome-scale metabolic network reconstructions of five life cycle stages of *Plasmodium falciparum (P*. *falciparum)*. We used these stage-specific genome scale metabolic models GeMMs to characterize functional metabolic features of each stage as well as to predict essential targets whose inhibition would interfere with malaria growth across asexual, sexual (transmission) and mosquito stages. Moreover, we reconstructed GeMMs for four additional Plasmodium species that infect rodents, non-human and human primates (including *P*. *vivax*, *berghei*, *cynomolgi*, and *knowlesi*). We used these Plasmodium GeMMs to investigate cross-species similarities and differences with particular focus on characterizing functional metabolic differences between rodent- and human-infecting species. Results provide a means to rank order and stratify established and new malaria treatment targets in addition to providing key insights into differences between rodent versus primate specific infections and implications for the interpretation of experimental animal models.

## Results

### Reconstructing and validating the metabolic network of *P*. *falciparum*, *i*AM-Pf480

**Fig 1:**
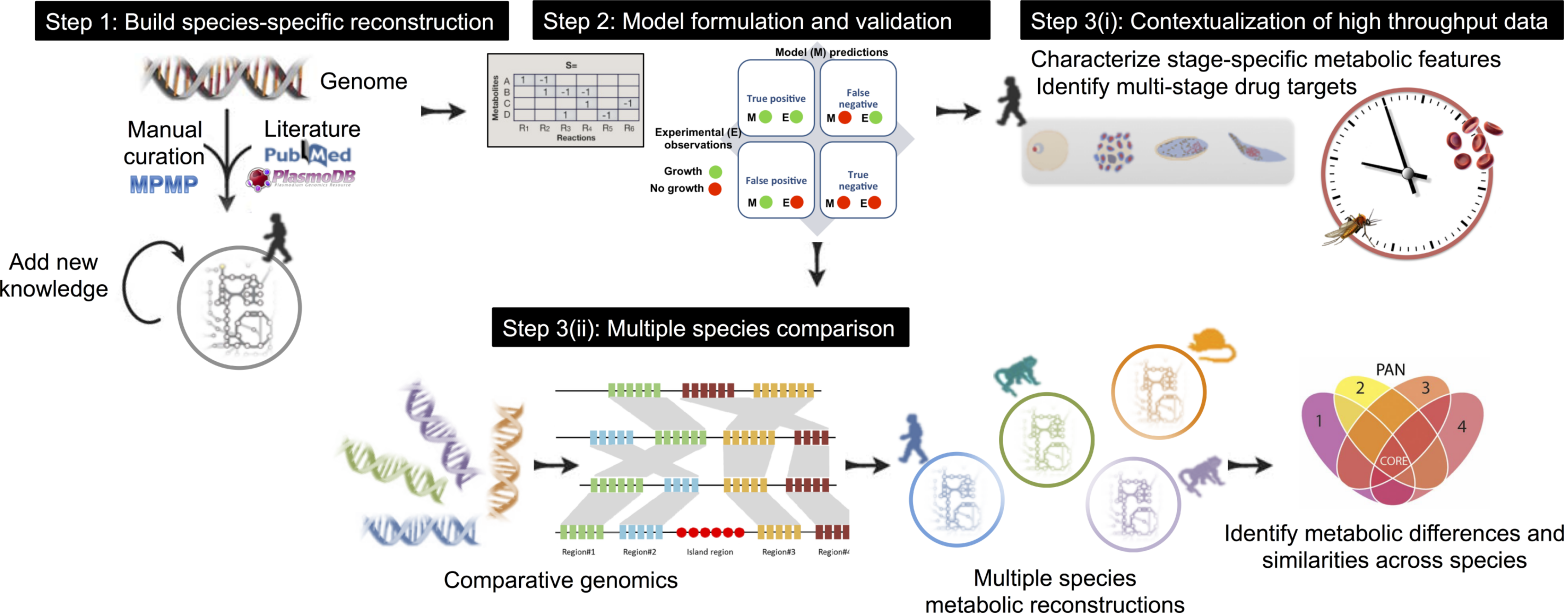
The workflow for manually curated, data-driven multi-species reconstructions of a representative fraction of the plasmodium genus. Step 1. The Plasmodium falciparum reconstruction was built using the genome annotation, lists of biomolecules, the literature, and organism-specific databases. The reconstruction was refined through iterations of manual curation, hypothesis generation, validation against experimental data and incorporation of new knowledge[15]. Step 2. The reconstruction was converted into a model by specifying inputs, outputs and relevant parameters, and by representing the network mathematically. The model was validated against omic and physiological data. Step 3(i). Once networks were accurately reconstructed and converted into stage-specific *in silico* models, using high throughput data, the stage-specific models were then used to predict genes critical for growth in different life-cycle stages and to detect stage-specific redirection of flux in the parasite’s central carbon metabolism. Step 3(ii). Comparative genomics and manual curation were used to build reconstructions for Plasmodium species commonly used in experimental animal models to test how predicted drug targets differ from predictions in the human-infecting system. Multi-species metabolic models were also used to characterize pan and core metabolic capacities of the different species.

**Fig 2:**
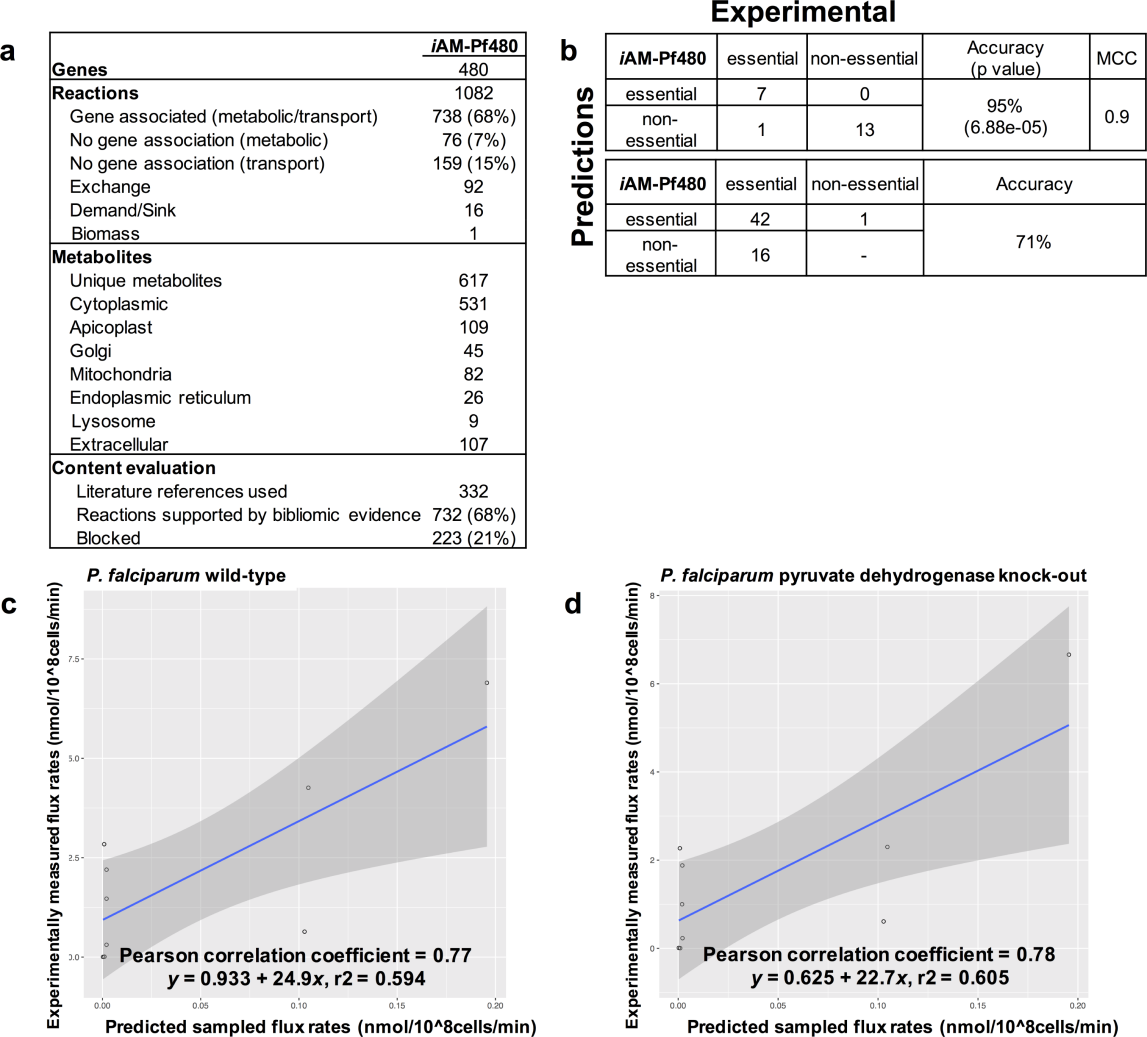
Content description and performance evaluation of *i*AM-Pf480 (*P*. *falciparum*). **(A)** Reaction and gene content of *i*AM-Pf480 (see details in Table A in S1 Text). **(b)** Comparison of *i*AM-Pf480 gene essentiality predictions (simulating standard *in vitro* growth conditions, Table B and C in S1 Tables) showed 95% and 71% accuracy when compared to single gene deletion and drug inhibition experiments, respectively. *In silico* gene essentiality was graded according to the percentage of reduction in growth rate compared to wild type. Fisher exact test as well as Mathew correlation coefficient (MCC) were used to compute significance of overlapping consistent predictions for *i*AM-Pf480. (C-D) Validation of iAM-Pf480 predicted glycolytic flux rates against experimentally measured fluxomic data[20] in **(c)** wild-type and **(d)** PDH-knock out *P*. *falciparum* while simulating standard *in vitro* growth conditions. Flux rates are in nmol/ 1x10^8^ cells/min.

A manually curated and quality-controlled[15] metabolic network reconstruction of *P*. *falciparum* (Fig 1, Step 1), *i*AM-Pf480, was constructed to interrogate the parasitic metabolic capabilities throughout the life cycle stages of malaria. *i*AM-Pf480 (Fig 2A) was built using the genome annotation of *P*. *falciparum* (Plasmodb.org, v24), the Malaria Parasite Metabolic Pathway (MPMP) Database (http://mpmp.huji.ac.il/), and specific biochemical and genetic characterization studies from 332 primary and review literature reference articles (Table A in S1 Tables). The metabolic network of the *P*. *falciparum* accounts for 1083 reactions, 617 unique metabolites and 480 genes localized to their respective intracellular compartments and organelles, including the cytoplasm, mitochondrion, the plastid-like apicoplast, endoplasmic reticulum, Golgi apparatus, and lysosome. Gene-protein-reaction (GPR) associations could be defined for 480 genes and 68% of all enzymatic reactions (Fig 2A).

### *i*AM-Pf480 accurately predicts gene essentiality and internal flux rates

In order to validate (Fig 1, Step 2) *i*AM-Pf480 predictions, we tested if *i*AM-Pf480 could correctly predict gene essentiality (Fig 2B). To accomplish this, we compiled a list of experimentally confirmed gene knockouts (n = 21, Table B in S1 Tables) and phenotypes resulting from targeted inhibition of enzymatic activities with drugs (n = 59, Table C in S1 Tables) in *P*. *falciparum*[16]. Under standard growth conditions, *i*AM-Pf480 correctly predicted 95% and 71% for the single gene knockouts and drug inhibition phenotypes, respectively (Fig 2B, Table B and C in S1 Tables). We also compared *i*AM-Pf480 gene essentiality predictions to *i*TH366[17], *i*Pfa[18] and *i*Pfal17[19], using our set of experimentally validated targets (Table B and C in S1 Tables), which revealed that *i*AM-PF480 accounts for a larger scope of genomic content, a larger biochemical complement, and functionally outperformed previously published *P*. *falciparum* models (see supplementary material, Fig A and Table L in S1 Text, Table B and C in S1 Tables).

*i*AM-Pf480 flux predictions were validated against available rapid stable-isotope labeling data to assess the metabolic flux changes in glycolysis of wild-type (WT) versus apicoplast pyruvate dehydrogenase knockout (PDHapi-KO) *P*. *falciparum* parasites[20]. Glucose and hypoxanthine uptake rates[21,22] were used to constrain the model (Table D and E in S1 Tables). Model-predicted glycolytic flux rates for both WT and PDHapi-KO showed good correlation (Pearson correlation coefficients 0.77 and 0.78 for WT and PDHapi-KO, respectively) with published experimentally measured fluxomic data[20] (Fig 2C and 2D).

Validation through fluxomic, single gene knockout, and drug targeting enzymatic assays provided confidence in the content and predictive capabilities of the metabolic model, setting the stage for further investigation into the consequences and capabilities of the parasites metabolic architecture (Fig 1, Step 3).

### Metabolic characteristics of *P*. *falciparum* life cycle stages

**Fig 3:**
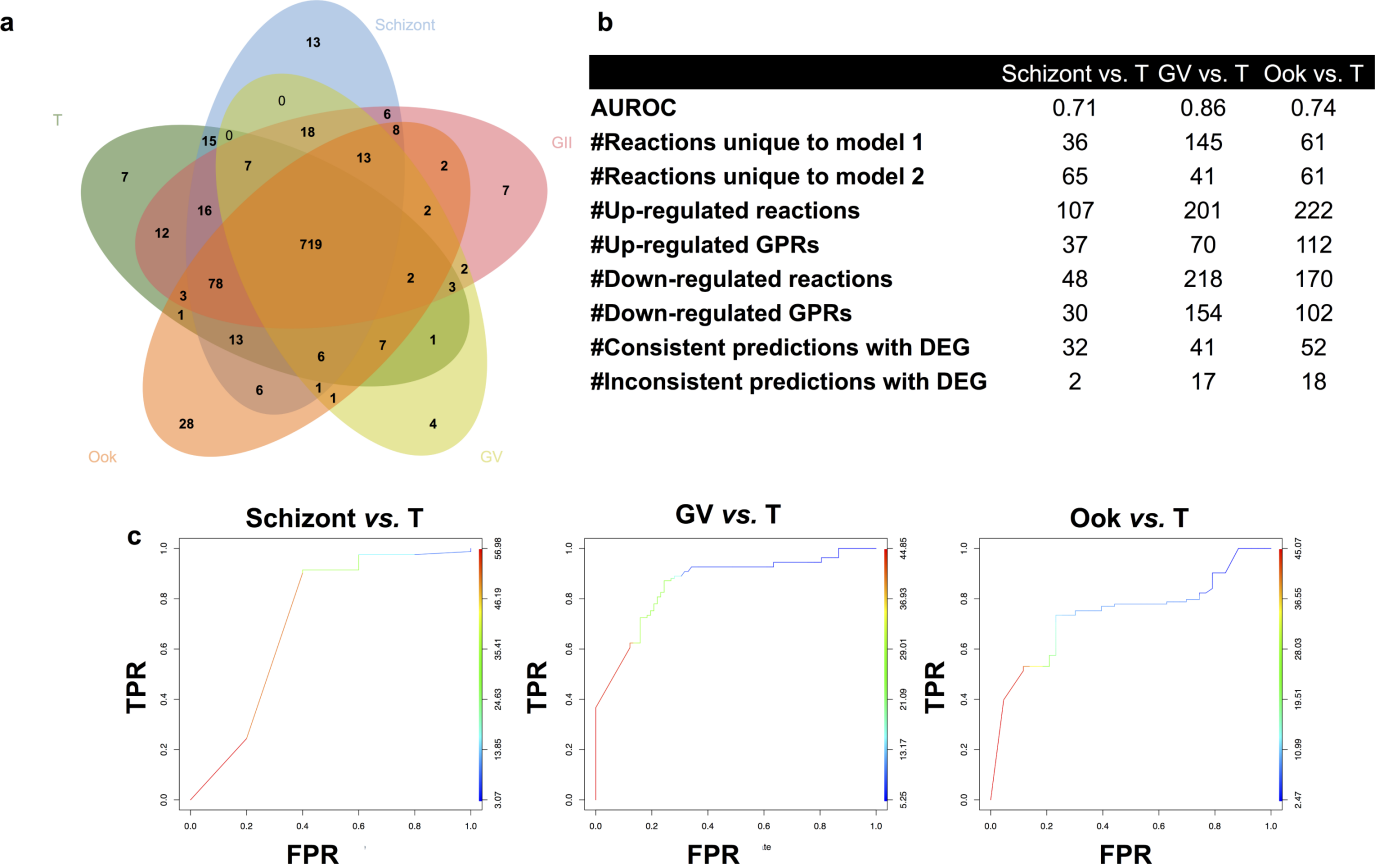
Life cycle stage specific models of *P*. *falciparum* predict gene targets that are essential for asexual, sexual and mosquito stages. **(a)** The core and pan metabolic content of the genome-scale life cycle stage specific models of *P*. *falciparum* are 720 and 1002 reactions, respectively. **(b-c)** Parameters and AUC plots for performance evaluation of stage-specific pairwise differential gene expression comparisons following a similar approach to Nam, et al.[52] (S2 Fig). AUROC: area under the receiver operator curve (ROC), DEG: differential expression analysis, T: trophozoite, GII: early gametocyte stage, GV: late gametocyte stage, Ook: ookinete.

Only approximately 1% of the asexual parasites develop into male and female gametocytes in response to yet unknown cues[23]. However, most current therapies are targeted against the blood stages, which result in clinical infections[2]. Thus, there is a pressing need to investigate potential targets that are critical for both the asexual and gametocyte stages to suppress both malarial transmission and active infection[23]; identification of such targets require understanding the stage-specific metabolic capabilities of *P*. *falciparum*. Towards this end, stage-specific models of metabolism throughout malaria’s life cycle were constrained using multiple data types (Fig 1, step 3i). Stage-specific growth rates[24], glucose and lactate secretion rates[25], as well as stage-specific transcriptomic data[26] were used to constrain *i*AM-Pf480 producing five distinct stage-specific models; trophozoite (T), schizont, early gametocyte (GII), late gametocyte (GV) and ookinete (ook) (Fig 3A) (see Table F in S1 Tables and Methods for details). In the early gametocyte stage (GII), biomass precursors production was permitted; however in the late, metabolically quiescent[23,25] mature gametocyte stage (GV), ATP generation was optimized[27] without associated net biomass accumulation.

**Fig 4:**
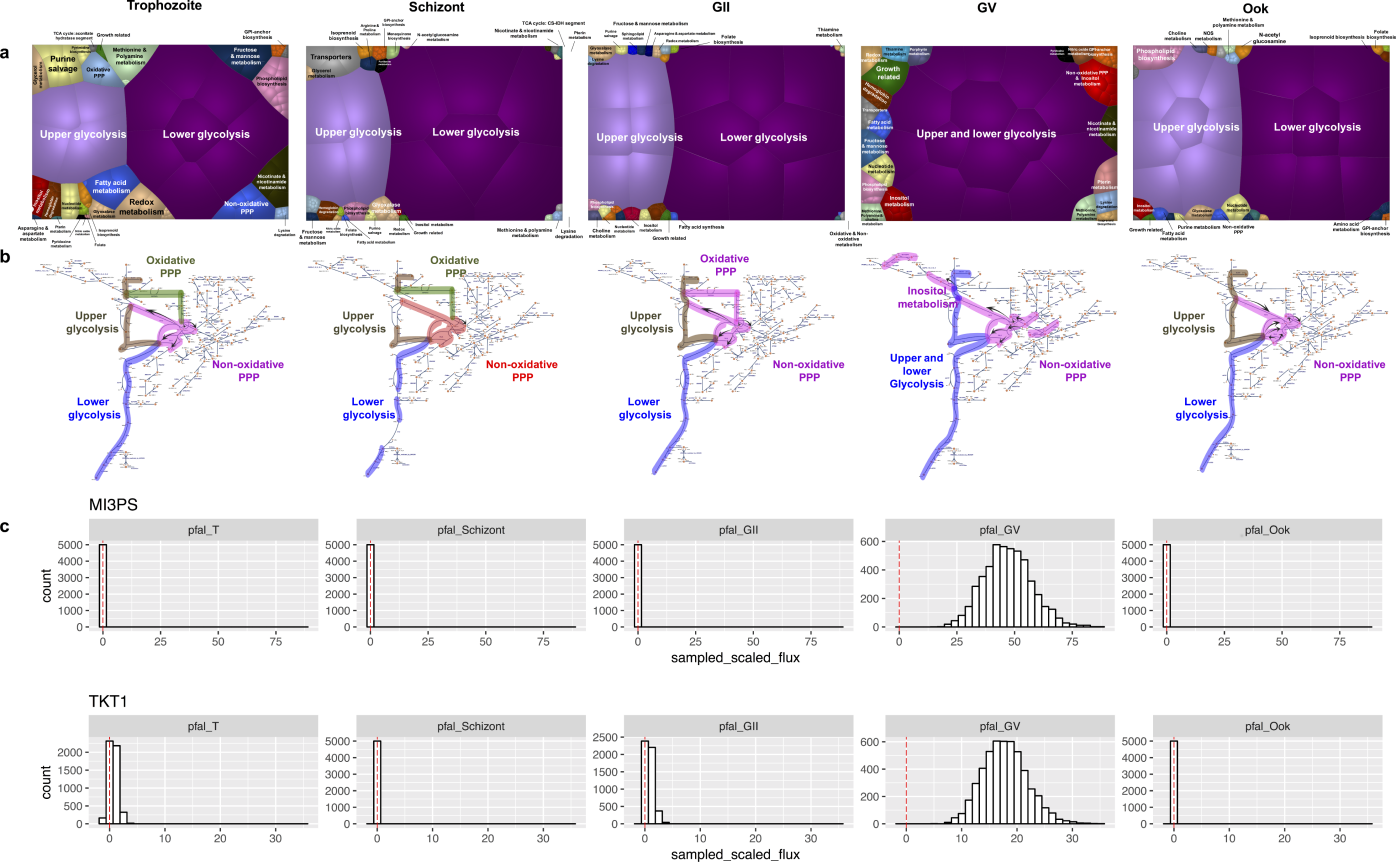
Stage-specific central metabolic flux patterns in malaria. **(a)** Correlated reaction sets for iAM-Pf480 were used to define stage and model specific pathways, which were analyzed and compared across different stages. Modularity indices (see methods) were 0.023, 0.024, 0.026, 0.145, 0.022 for the T, Schizont, GII, GV, and Ook stages, respectively. In proliferative versus non-proliferative stages of malaria, there were changes in the patterns of central carbon metabolism, notably the non-oxidative PPP and glycolysis. **(b)** The direction of flux in the non-oxidative branch of PPP goes towards production of glycolytic intermediates in the Trophozoite, Schizont, GII, and GV stages but not the Ookinete stage. Reversal of non-oxidative pentose phosphate pathway fluxes in the Ook enables provision of ribose 5 phosphate (r5p) needed for the synthesis of nucleotide precursors of DNA. The non-oxidative branch in the schizont is colored in red indicating its coupling to growth rate in this stage (Table G in S1 Tables). Both the oxidative and non-oxidative PPP branches were correlated in GII. Glycolysis was split into upper and lower branches in all stages except GV where the non-oxidative PPP branch was correlated with inositol metabolism. Arrows are omitted from the schizont pathway map to account for reduced flux values relative the other 4 stages. **(c)** Predicted sampled flux distribution are shown in the non-oxidative branch of PPP (Transketolase; TKT1) and inositol metabolism (myo-inositol-3-phosphate lyase; MI3PS) across all the stages showing increased involvement of inositol metabolism in the GV stage (see supplementary material for discussion and S1 Fig for the high resolution version of the figure).

Generic, canonical reaction groupings into pathways do not inform functional states. However, GeMM-based simulations can be used to calculate groups of reactions with highly correlated metabolic fluxes under a set of condition(s), i.e., correlated reaction sets (co-sets), that in turn can yield insight into metabolic capabilities[28,29] and also reduce network size into functionally correlated modules of reactions. Artificial Center Hit and Run[30] sampling was used to determine the steady state flux distributions of the stage-specific models, and these were used to compute co-sets across different conditions (Table G in S1 Tables) (see Methods). 612 reactions were involved in co-sets with 3 or more reactions spanning pathways related to anabolic and catabolic processes for amino acids, fatty acids, and biomass production (Table G in S1 Tables). A quantitative assessment of flux magnitude as well as co-set size across the different stages are visualized as Voronoi plots (Fig 4A). Comparison across the different stages reflects that the biomass constraint provides the greatest influence on the size and modularity of the metabolic network, thus the late gametocyte, with the most relaxed biomass constraint, had the most modularity where high modularity reflects networks with co-sets that are similar in size, whereas low modularity refers to networks that have one or two co-sets that are very large in comparison to the rest of the co-sets in the network (Fig 4).

### Stage-dependent redirection of central metabolic carbon flux in malaria

Across the different stages, there were considerable changes in central carbon metabolism, based on the co-sets (Fig 4). The metabolic simulations predicted splitting of glycolysis into independent upper and lower branches to divert biomass (nucleic acids, lipids, glycosylated proteins) required for proliferating parasite stages, whereas the late gametocyte stage is non-proliferative, accordingly, both the upper and lower glycolytic branches were in one co-set. Contrary to what was believed about the pentose phosphate pathway (PPP) deploying the oxidative arm only during the early stages of the parasite intra-erythrocytic development cycle (IDC) and the non-oxidative arm in the later stages of the IDC [31], simulations with stage-specific models showed that: 1) both the oxidative and non-oxidative arms were active with low fluxes in the early stages (trophozoite, schizont and GII), 2) the non-oxidative arm operated in the backward direction to produce glycolytic intermediates in the early stages while the oxidative arm produced NADP+ and ribose-5-phosphate (r5p), and 3) only the non-oxidative branch of PPP was active in the GV stage in the backward direction and was correlated with inositol metabolism (Fig 4) underscoring the importance of inositol metabolism across the transmissible stage of malaria[32] 4) similar to the GV stage, only the non-oxidative arm was active in the ookinete stage, albeit in the forward direction using fructose-6-phosphate (f6p) and glyceraldehyde-3-phosphate (g3p) supplied by glycolysis, thus maximizing the production of r5p (Fig 4B).

### Multi-stage evaluation of experimentally validated single stage deletions across the entire organism

**Fig 5:**
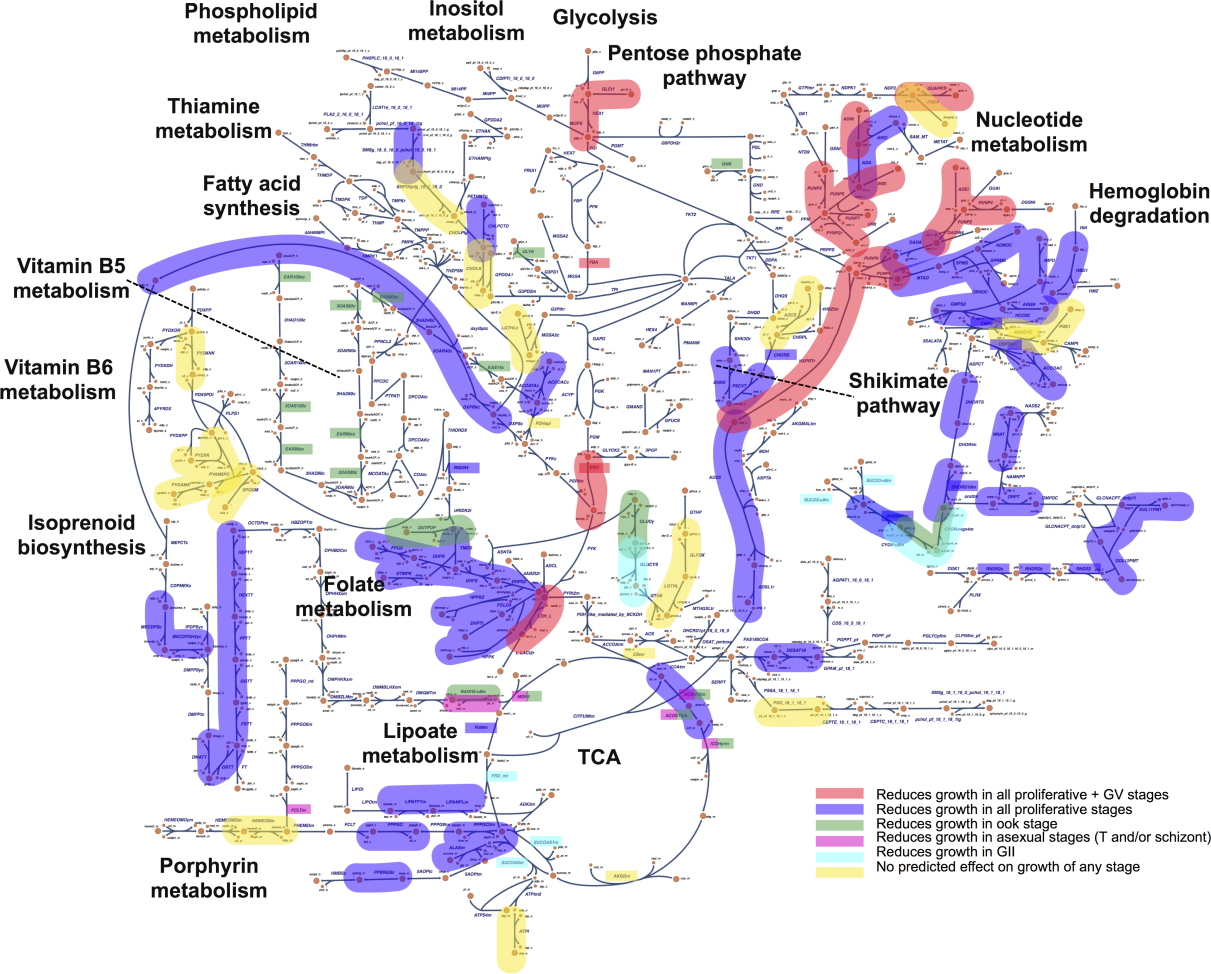
Stage-specific essentiality predictions of experimentally validated druggable targets and single-gene deletion experiments. Comprehensive map of experimentally tested treatment targets for *P*. *falciparum* with stage-specific model predictions projected (in color) projected on top of the map. Colored reaction pathways correspond to drug inhibition studies (Table C in S1 Tables) and colored reaction names in rectangles correspond to single gene deletion experiments (Table B in S1 Tables). The color legend inset corresponds to iAM-Pf480 predictions. Validated drug targets that are also predictive to reduce growth in proliferative as well as late gametocyte stages are of particular interest. This comprehensive assessment of the model and experimental results enables stratification of existing drugs, new drugs to target, as well as new areas of metabolism warranting further investigation. See S2 Fig for the high resolution version of the figure.

We constructed a comprehensive map of druggable targets for *P*. *falciparum* (Fig 5) using our curated list of experimentally validated targets (Table B and C in S1 Tables). This was used to compare to predictions made by the stage-specific *P*. *falciparum*. Selecting *stage-specific* targets spanning the parasite’s life cycle could promote the design of strategies for potential multi-stage targets or combination of existing drugs. The color scheme of highlighted reactions denotes model prediction classification across all stages. The red group in Fig 5 highlights reactions sensitive in the proliferative stage as well as the late gametocyte (GV) stage; this is of particular importance since the late gametocyte is of high clinical interest to target and represent a large percentage of the parasitic load that is not targeted by some of the more commonly used treatment drugs.

Reactions associated with genes from experimentally validated single gene targets (Fig 2B) are highlighted in the colored rectangles in Fig 5. There are several gene deletion associated reactions for which drugs have not been developed; these highlight potential targets for new drug development. We also note that the yellow group identifies reactions that were missed by the models, and highlights areas for future model refinement. By overlaying stage-specific model predictions on top of the experimental (single-stage) validated drug targets, the network map aids in the prioritization of drug target characterization.

### Systems analysis of metabolism across the *Plasmodium* genus

**Fig 6:**
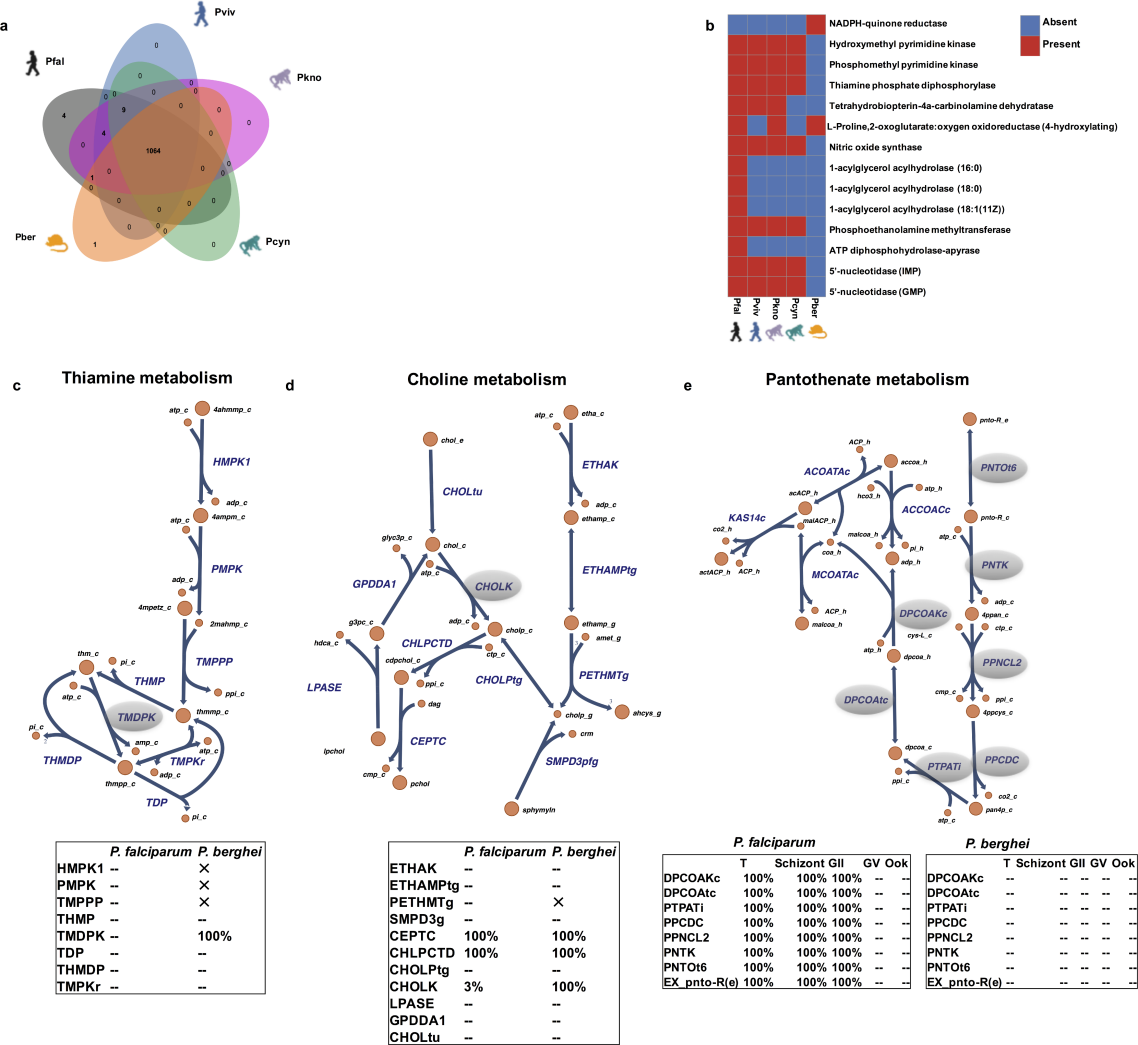
Species-specific models provide mechanistic explanation for differences in drug response between human- and rodent-infecting malaria species. **(a)** The core and pan metabolic content of 5 malaria species was identified based on the respective species-specific reconstructions. The core content, illustrated by the intersection of the Venn diagram, is shared by all species. The pan content represents the union of the content across all of the multi-species reconstructions. **(b)** 14 metabolic reactions differed in their presence across the 5 reconstructed Plasmodium species. **(c)** Thiamine pyrophosphokinase (TPK) and **(d)** Choline kinase (CK) were predicted by the models to be essential for the growth of the rodent-infecting species (*P*. *berghei*) while their deletion had no effect on the growth of human and non-human primate species. Differential essentiality of TPK is due to absence of phosphomethylpyrimidine kinase and thiamine-phosphate pyrophosphorylase the rodent-infecting species. In the case of CK, the differential essentiality is due to the absence of phosphoethanolamine N-methyltransferase. (See Table A in S1 Tables for reactions abbreviations and gene-protein-reaction associations). **(e)** Pantothenate metabolism showed differences in essentiality between stage- and species-specific models. Tables indicate percentage in growth reduction compared to the WT upon deletion of the respective gene. ‘X’ indicates absence of a reaction from the respective reconstruction, ‘—‘ indicates no effect on growth upon deletion of the corresponding reaction and ‘%’ indicates the growth reduction percentage resulting from deletion of the corresponding gene. T: trophozoite, GII: early gametocyte stage, GV: late gametocyte stage, Ook: ookinete. See S3 Fig for the high resolution version of the figure.

The use of experimental model organisms, such as mice, has yielded a wealth of knowledge about the Plasmodium genus. However, there has been relatively limited investigation into species-specific differences in Plasmodium metabolism[33–35]. While there is 94% (422/448) homology among the metabolic genes of the different species (Table H in S1 Tables), it is unclear how they differ in their metabolic capabilities. Further, differences have been observed between rodent- and human-malaria infecting species to certain drug inhibitors[36] but no mechanistic explanation was attributed to these differences. Beginning with the *i*AM-Pf480 GeMM we systematically studied the functional metabolic differences between 5 different Plasmodium species (Fig 1, Step 3ii): *Plasmodium falciparum* 3D7 (Pfal), *Plasmodium vivax (Pviv) Sal-1 (i*AM-Pv461*)*, *Plasmodium berghei (Pber) ANKA (i*AM-Pb448*)*, *Plasmodium cynomolgi (Pcyn) strain B (i*AM-Pc455*)*, and *Plasmodium knowlesi (Pkno) strain H (i*AM-Pk459*)*. The core metabolic content reflects the intersection of the genes, reactions, and metabolites of all five reconstructions, whereas the *pan metabolic content* is the union of these entities. The core Plasmodial metabolic content is comprised of 1064 reactions and 422 orthologous genes (Fig 6A) and the pan metabolic capabilities include 1083 reactions corresponding to 448 orthologous genes (Table H in S1 Tables), reflecting a considerable level of conservation. However there are multiple functional differences cross the metabolic GeMMs (Fig 6B, Table H and I in S1 Tables). The differences in metabolic reaction content across the five reconstructed species predominantly involve co-factor metabolism (4 reactions), phospholipid metabolism (4 reactions), and purine/pyrimidine metabolism (3 reactions) (Fig 6B).

We performed *in silico* single gene deletion analysis for the set of 448 orthologous genes shared among the five species (Table H in S1 Tables). The deletion of 15% (67/448) of these orthologous genes caused a 100% reduction in growth across all species (Table H in S1 Tables). These genes spanned several metabolic subsystems with the majority involved in isoprenoid biosynthesis, phospholipid metabolism, as well as purine and pyrimidine metabolism. Interestingly, 19 genes out of this set have already been targeted by drug inhibitors (Table C in S1 Tables) while the remaining 48 orthologous genes represent overlooked novel druggable vulnerabilities in malaria.

### Variations in thiamine and choline metabolism in rodent versus non-rodent infecting species

14% (61/448) of the orthologs differed in their essentiality across the 5 Plasmodium species. Reactions with the most striking differences in essentiality across malaria species were between the rodent and non-rodent species, namely: thiamine pyrophosphokinase (TPK), and choline kinase (Table H in S1 Tables). Both genes were predicted to be essential in *P*.*berghei* only, while their deletion had no effect on growth in any of the non-rodent species.

Thiamine analogs[36] and choline kinase (CK) inhibitors[37] have been tested as antimalarial therapeutics both in plasmodium species that infect human (*in vitro*) and rodents (*in vivo*); however, it is not clear from these studies whether an equally potent antimalarial effect is observed in both species. Our multi-species reconstructions revealed three key thiamine (Vitamin B1) biosynthesis enzymes: phosphomethylpyrimidine kinase (PMPK), hydroxyphosphomethylpyrimidine kinase (HMPK1), and thiamine-phosphate pyrophosphorylase (TMPPP) that are absent in rodent malaria, but present in non-rodent malarial species (Fig 6C). Consequently, TPK was predicted to be essential for rodent malaria, but non-essential in non-rodent species, which can replenish thiamine pyrophosphate through the HMPK1-PMPK1-TMPPP pathway (Fig 6C).

*In silico* deletion of choline kinase (CK), the first enzyme in the Kennedy pathway (CDP-choline pathway) for synthesis of phosphatidylcholine (PC) (Fig 6D), inhibited growth of the rodent species while causing only marginal reduction in growth (3%) in the primate and human species. The model simulations revealed the difference in essentiality of CK was the result of the lack of phosphoethanolamine N-methyltransferase (PMT) in *P*. *berghei*, which in turn rendered it incapable of *de novo* PC synthesis from ethanolamine (Fig 6D). Thus, efforts to perturb PC for malaria treatments will require different strategies in human species than in non-human-infecting species since decreased potency is expected when perturbing choline metabolism in the non-rodent relative to rodent-infecting species.

### Differential pantothenate dependency in human versus rodent asexual stages of malaria

A comparative analysis of stage-specific models of human and rodent species (see Methods and supplementary material for details), based on co-sets and *in silico* single gene deletion experiments, showed that pantothenate metabolism was not essential for growth in any of the life cycle stages of *P*. *berghei*. In contrast, pantothenate metabolism was essential for growth during the asexual and early gametocyte stages of *P*. *falciparum* (Fig 6E, Table K in S1 Tables). In line with recent evidence[38], our stage- and species-specific models predicted that the pantothenate transporter activity was essential in human malaria, but was mostly dispensable in rodent parasites. Pantothenate is a precursor of the enzyme cofactor coenzyme A (CoA) and the capability of *de novo* synthesis of CoA distinguishes *P*. *falciparum* asexual forms from its sexual counterparts as well as from the rodent and avian malaria parasites, thus challenging the assumption that rodent and human malaria parasites utilize similar nutrient acquisition strategies[39].

## Discussion

Therapeutic drugs that target multiple stages of the parasite, including sexual and asexual stages, will facilitate eradication of malaria. Developing effective medications will require understanding basic biological mechanisms, particularly the limitations of experimental animal models that are used as surrogates for understanding human Plasmodium pathogenesis. Systems analysis are thus needed to interpret and integrate multiple, large disparate datasets to unravel the complex life cycles of these pathogens.

In this study we use genome-scale metabolic modeling to interrogate malaria stage- and species-specific metabolic capabilities. Through the integration of high-throughput data, careful manual curation, and model prediction validation, we reconstructed detailed stage-specific models that span five distinct stages of the life cycle of *P*. *falciparum*. Since GeMMs allow condition-specific analyses, we were able to simulate the effects of reaction inhibition across different stages of the parasite’s life cycle and to identify drugs that are effective across more than one stage. Moreover, we detected stage-dependent metabolic redirection of flux in central carbon metabolism of the parasite that stage-matched proliferation requirements.

The overall genome organization and content across Plasmodium species is highly conserved, with about 4000 conserved syntenic genes located within the central core regions of the 14 chromosomes[40]. Subsequently, it is frequently assumed that findings from the animal models will directly translate to the human-infecting species, particularly in areas of essential, core metabolism, given the high degree of homology across the malaria species (94%). However, our GeMMs for multiple Plasmodium species highlight important metabolic differences. The GeMM simulations provide a mechanistic explanation for why *P*. *berghei* would be more sensitive to thiamine analogs as well as to drugs interfering with PC metabolism[36] and not in human infecting parasites. Additionally, differences in pantothenate metabolism were revealed in stage-specific analysis of *P*. *falciparum* and *P*. *berghei*, further highlighting potential differences between the metabolism of human- versus rodent-infecting species. This finding suggests a potential use of an auxotrophic mutant of *P*. *falciparum* defective in the *de novo* biosynthesis of pantothenate for vaccination in analogy to *Mycobacterium tuberculosis*[41].

These multi-species GeMMs have enabled us to make informed predictions about specific differences between rodent and non-rodent metabolic capabilities, underscoring the fact that the metabolic architecture and nutritional requirement of a rodent malaria species does not necessarily predict that of a human malaria species[39]. Furthermore, in addition to using the models to make predictions and gain systems-level insights to malaria metabolism in relation to drug targeting, these models can be used as a foundational structure upon which additional high-throughput data can be analyzed and predictive simulations can be conducted, thus leading to improved understanding, testable hypotheses and increased knowledge[42].

## Materials and methods

The methods employed for the reconstruction, simulation, and analyses presented in this manuscript are briefly summarized below, with further details regarding the procedures, protocols, calculations, and quality control measures provided in the supplementary material. All models are available as part of the supplementary material and will be deposited in the BiGG[43] database.

### iAM-Pf480 network reconstruction and constraint based models

The genome sequence and genome annotations for *P*. *falciparum* were downloaded from Plasmodb.org (release 26). A list of *P*. *falciparum* metabolic pathways was built based on current genome annotation of *P*. *falciparum* (Plasmodb.org), the Malaria Parasite Metabolic Pathway (MPMP) Database (http://mpmp.huji.ac.il/), and malaria-specific biochemical characterization studies (Table A in S1 Tables). The stoichiometric matrix was constructed with mass and charge balanced reactions in the standard fashion and flux balance analysis was used to assess network characteristics and perform simulations[44]. Linear programming calculations were performed using Gurobi (Gurobi Optimization, Inc., Houston, TX) and MATLAB (The MathWorks Inc., Natick, MA) with the COBRA Toolbox[45,46].

### Validation of iAM-Pf480 predicted glycolytic flux rates and single gene deletion essentiality predictions

We tested *i*AM-Pf480-predicted flux rates against kinetic flux data (rapid stable-isotope labeling) of glycolysis in wild-type (WT) and pyruvate dehydrogenase (PDH) deficient *P*. *falciparum* parasites cultured *in vitro*[20]. The generic *i*AM-Pf480 model was allowed to uptake metabolites available in standard *in vitro growth* conditions (RPMI 1640, 25 mm HEPES, 2 mm l-glutamine supplemented with 50 μm hypoxanthine and 10% A+ human serum[20]) (Table D in S1 Tables). Uptake rates for glucose and hypoxanthine were obtained from literature[21,22].

For validation of *in silico* single gene deletion essentiality predictions, we compiled a curated list of experimentally validated gene knock-outs (n = 21, Table B in S1 Tables) and phenotypes resulting from targeted inhibition of enzymatic activities with drugs (n = 59, Table C in S1 Tables) in *P*. *falciparum* based on our recently published list[16] of targeted chemical compounds in MPMP.

### Life cycle stage specific models of *P*. *falciparum*

An experimentally measured growth rate (lower bound of 0.045 mmol/gDW/h corresponding to approximately 15 hours[24] was imposed on the biomass function). Lactate secretion for asexual stages (93% of uptake glucose) was applied to the *i*AM-Pf480 model simulating *in vitro* growth conditions (see fluxomics data section). For the gametocyte stages, we developed two models. The first model (GII) simulated early gametocyte stage II which is metabolically active and hence, the objective function was set to maximize the production of biomass precursors. The constraint on the lower bound of the biomass function was relaxed to 0 mmol/gDW/h since it’s expected that the early gametocyte stages will exhibit a lower growth rate compared to the asexual stages. Lactate secretion was set to a minimum of 80% of glucose uptake rate[25]. The second model (GV) represents mature, metabolically quiescent gametocyte stages. The objective function in the GV model was to set to optimize ATP production[23] and while no flux was allowed in the biomass function (lower bound = 0 and upper bound = 1e-9). Uptake of N-acetyl glucosamine (GlcNAc) was allowed in both gametocyte models since GlcNAc induces gametocytogensis[47]. For the ookinete model, the glucose uptake was constrained to 10% of the asexual stages glucose uptake rate since the mosquito gut is a glucose-rare environment[25].

*P*. *falciparum* 3D7 life cycle stage-specific RNA-Seq data was downloaded from SRA archive (SRP009370)[26]. SRA files were converted to fastq files using the sra-toolkit[48]. Tophat2[49] was used for the alignment (—library-type fr-unstranded) libraries. PICARD (http://broadinstitute.github.io/picard/) and samtools[50] were used for processing the aligned reads and HTSeq[51] was used to produce read counts (—stranded = no). The normalized read counts were then used to further constrain the stage-specific models (Fig B in S1 Text).

Validation in part was performed from growth rate predictions of overall biomass production rates in each life cycle stage of *P*. *falciparum* (Table F in S1 Text). The predictions showed an overall qualitative agreement with the experimentally observed growth phenotypes of the parasite during the asexual and sexual stages. Specifically, our models successfully predicted significant increase of the growth rate (FDR < 0.05) by 1.8 and 20 fold in the trophozoite relative to the early and late gametocyte stages, respectively (Table F in S1 Text). The ookinete model was the only malaria stage-specific model that was able to grow in absence of glucose (although the reduction in growth was 93%), which is in line with the glucose-rare medium in the mosquito gut where this stage develops[25].

### Performance evaluation of stage-specific models against differential gene expression (DEG) analysis results

Stage-specific model predictions were compared against differential gene expression (DEG) following a previously published workflow[52], outlined in Fig B in S1 Text. Briefly, differential gene expression analysis was carried out between every two stages and the lists of significantly differentially expressed genes (FDR < 0.05 and (> 75^th^ or < 25^th^ percentile of the log2 fold change in expression)) were later used for evaluation of stage—specific models’ predictions. The network flux states were sampled and significantly different reactions (FDR < 0.05 and (> 75^th^ or < 25^th^ percentile of the log2 fold change in reaction fluxes)) were identified following removal of loop reactions. The corresponding genes were selected using gene-protein-reaction relationships and were compared against the list of significantly differentially expressed genes.

### Co-sets

Correlated reaction sets (co-sets) were calculated using the sampled steady state solution points for the iAM-Pf480 stage-specific models (COBRA toolbox[46] ‘identifyCorrelSets’ with a correlation cutoff threshold of 0.95). Only co-sets containing 3 or more reactions were labeled, since these co-sets generally represent transport of individual metabolites and not biochemical pathways *per se*. Sampled reaction fluxes in the pentose phosphate pathway were compared across the different stages and differential flux activity was acknowledged if the flux distributions were significantly different following multiple hypothesis correction, as previously described[52]. The modularity of the co-sets was assessed using the ratio of the mean size of co-sets divided by the maximum size of the co-sets for each stage. Voronoi plots were generated using TreeMap (v. 3.8.3) using the co-sets annotation (Table G in S1 Tables) and sampled flux distribution of each reaction in the corresponding co-set.

### Species-specific model reconstruction

Genome-scale metabolic models were reconstructed for five Plasmodium species (*P*. *falciparum* ‘Pfal’, *P*. *knowlesi* ‘Pkno’, *P*. *vivax* ‘Pviv’, *P*. *cynomolgi* ‘Pcyn’, and *P*. *berghei* ‘Pber’). The details of the procedure for building Plasmodium multi-species genome-scale metabolic models are outlined in (Fig C in S1 Text).

## Supporting information

S1 Text Supporting information description. iAM-Pf480 Network reconstruction and refinement
Generation of the biomass objective function.
Model naming convention
Refinement of iAM-Pf480.
Validation of iAM-Pf480 predicted glycolytic flux rates.
Performance evaluation and validation of iAM-Pf480 gene essentiality predictions
Comparison of iAM-Pf480 to previously published P. falciparum models
Comparison to iTH36610
Comparison to iPfa22
Comparison to iPfal1723
P. falciparum life cycle stage-specific model building and validation procedures.
Performance evaluation of stage-specific models against differential gene expression (DEG) analysis results.
Co-Sets predict a stage-dependent fate of glucose-6-phosphate in P. falciparum
Species-specific model building procedure
Reconstruction of species-specific models.
Curation notes for specific enzymes that differed across the species.
Host-specific hemoglobin composition
Validation of the species-specific reconstruction workflow
Metabolic Similarities across Plasmodium species
Variations in metabolic capacities between rodent- and human-infecting malaria species
Phylogenetic analysis using TPK and CK vs. DHODH.
Life cycle stage-specific models of P. berghei
References (Supporting Information)
(PDF)

S1 Tables Supporting tables. Table_SA iAM-Pf480 content description
Table_SB Single gene deletion list and performance evaluation of iAM-Pf480 and iTH366
Tables_SC Druggable targets compiled list and performance evaluation of iAM-Pf480 and iTH366
Tables_SD Validation of iAM-Pf480 predicted flux rates: uptake rates used to constrain and validate model predictions as published in PMID:24163372
Tables_SE Validation of iAM-Pf480 predicted flux rates: correlation between predicted and experimentally measured flux rates for wild-type and pyruvate dehydrogenase (PDH) deficient P. falciparum parasites
Table_SF Doubling time (h) times and glucose uptake rates of P. falciparum stage-specific models
Table_SG Correlated reaction sets (CoSets) across the life cycle stage-specific models of P. falciparum
Table_SH Gene essentiality predictions across the 5 plasmodium species studied in this work. Representative genes belonging to the same OG group are shown in the table. Pfal: P. falciparum, Pber: P. berghei, Pkno: P. knowlesi, Pcyn: P. cynomolgi, Pviv: P. vivax, grRed: growth reduction
Table_SI Variation in metabolic reactions between 5 plasmodium species: 14 enzymatic and transport reactions coded for by 9 genes that differe between species-models
Table_SJ Performance evaluation of iAM-Pb448
Table_SK Pantothenate CoSet and differential essentiality across P. falciparum and P.berghei stage specific models.
(XLSX)

S1 Fig High resolution version of main text Fig 4. (TIF)

S2 Fig High resolution version of main text Fig 5. (TIF)

S3 Fig High resolution version of main text Fig 6. (TIF)

S1 Model SBML and MATLAB files of the models described in this manuscript. (ZIP)
