# Supplementary material for: Functional interrogation of Plasmodium genus metabolism identifies species- and stage-specific differences in nutrient essentiality and drug targeting
Source: PLoS Comput Biol. 2018 Jan 4;14(1):e1005895. doi: 10.1371/journal.pcbi.1005895 (PMC5771636; doi:10.1371/journal.pcbi.1005895)
Supplement: S1 Text — iAM-Pf480 Network reconstruction and refinement Generation of the biomass objective function. Model naming convention Refinement of iAM-Pf480. Validation of iAM-Pf480 predicted glycolytic flux rates. Performance evaluation and validation of iAM-Pf480 gene essentiality predictions Comparison of iAM-Pf480 to previously published P. falciparum models Comparison to iTH36610 Comparison to iPfa22 Comparison to iPfal1723 P. falciparum life cycle stage-specific model building and validation procedures. Performance evaluation of stage-specific models against differential gene expression (DEG) analysis results. Co-Sets predict a stage-dependent fate of glucose-6-phosphate in P. falciparum Species-specific model building procedure Reconstruction of species-specific models. Curation notes for specific enzymes that differed across the species. Host-specific hemoglobin composition Validation of the species-specific reconstruction workflow Metabolic Similarities across Plasmodium species Variations in metabolic capacities between rodent- and human-infecting malaria species Phylogenetic analysis using TPK and CK vs. DHODH. Life cycle stage-specific models of P. berghei References (Supporting Information) (PDF) [file pcbi.1005895.s001.pdf]

SUPPORTING INFORMATION

Functional interrogation of Plasmodium genus metabolism identifies species and stage specific differences in nutrient essentiality and drug targeting

Alyaa M. Abdel-Haleem[1,2], Hooman Hefzi[3,4], Katsuhiko Mineta[1], Xin Gao[1], Takashi Gojobori[1], Bernhard O. Palsson[3,4,5], Nathan E. Lewis[3,4], and Neema Jamshidi*[6,7]

AUTHORS AFFILIATION

1 King Abdullah University of Science and Technology (KAUST), Computational Bioscience Research Centre (CBRC), Thuwal, 23955-6900, Saudi Arabia

2 King Abdullah University of Science and Technology (KAUST), Biological and Environmental Sciences and Engineering (BESE) division, Thuwal, 23955-6900, Saudi Arabia

3 Department of Bioengineering, University of California, San Diego, La Jolla, CA, USA

4 Novo Nordisk Foundation Center for Biosustainability at the University of California, San Diego School of Medicine, La Jolla, CA, USA

5 Department of Pediatrics, University of California, San Diego, La Jolla, CA, USA

6 Institute of Engineering in Medicine, University of California, San Diego, La Jolla, CA, USA

7 Department of Radiological Sciences, University of California, Los Angeles, CA, USA

* Corresponding Author, neema@ucsd.edu

Table of Contents

*i*AM-Pf480 Network reconstruction and refinement 2
- Generation of the biomass objective function. 2
- Model naming convention 2
- Refinement of *i*AM-Pf480. 2
- Validation of *i*AM-Pf480 predicted glycolytic flux rates. 4
- Performance evaluation and validation of *i*AM-Pf480 gene essentiality predictions 5

Comparison of *i*AM-Pf480 to previously published *P. falciparum* models 5
- Comparison to *i*TH366[10] 5
- Comparison to *i*Pfa[22] 6
- Comparison to iPfal17[23] 8

P. falciparum life cycle stage-specific model building and validation procedures. 9
- Performance evaluation of stage-specific models against differential gene expression (DEG) analysis results. 10
- Co-Sets predict a stage-dependent fate of glucose-6-phosphate in *P. falciparum* 11

Species-specific model building procedure 12
- Reconstruction of species-specific models. 12
- Curation notes for specific enzymes that differed across the species. 14
- Host-specific hemoglobin composition 15
- Validation of the species-specific reconstruction workflow 16
- Metabolic Similarities across Plasmodium species 17
- Variations in metabolic capacities between rodent- and human-infecting malaria species 18
- Phylogenetic analysis using TPK and CK *vs*. DHODH. 18

Life cycle stage-specific models of *P. berghei* 19
References 22

*i*AM-Pf480 Network reconstruction and refinement

Generation of the biomass objective function.

The biomass reaction for *P. falciparum* was built based on the cellular composition of *Leishmania major*, another protozoan parasite, as organism-specific information was unavailable [1]. DNA composition was calculated from the genome sequence. RNA composition was calculated from the frequency of bases across all CDS regions and amino acid composition from the frequency of use across all protein coding regions. The lipid composition of the parasite membranes was calculated from a previously published study [2]. The fatty acid contributions were calculated for those fatty acids with a > 5% contribution to the total phospholipid pool. Growth associated maintenance (GAM) requirements were assumed to be identical to *L. major*[1]. Additionally, metabolites with known roles but unknown percentage composition were added with a coefficient of 0.0001. This ensured that flux would be required through the corresponding biosynthetic pathways but not exert a noticeable effect on the growth rate [3]. The model accounts for six subcellular localizations: cytosol, mitochondria, Golgi apparatus, endoplasmic reticulum, food vacuole, and apicoplast, in addition to the extracellular compartment. Enzyme localization was determined through a combination of primary literature sources (Table A in S1 Tables) as well as computational tools such as PlasMit [4]. Following completion of the draft reconstruction, manual curation of the model was carried out to ensure that all biomass components could be produced which consisted primarily of evidence-based filling of metabolic gaps upstream of biomass components.

Model naming convention

We followed the previously established convention for naming genome-scale constraint-based models which mirrors the one already established for plasmids [5], i.e., "i" to denote "*in silico*," followed by the initials of the first author ("AM"), followed by the number of genes included in the model (e.g."480"). We have added the initials of each species (e.g. "Pf") to make it easier to differentiate the species-specific models.

Refinement of *i*AM-Pf480.

The resultant model was tested for accuracy in numerous ways. Briefly, the model was tested to ensure that it was unable to produce any biomass precursors when no external metabolites were provided. It was also unable to produce purine nucleotides without external purine sources being present since the parasite obtains these from the host. Lastly, the model was able to produce a full complement of biomass components when presented with a simulated *in vitro* environment (see validation of *i*AM-Pf480 predicted glycolytic flux rates).

The process of reconstructing metabolic networks is an iterative process [6] beginning with the initial creation of a draft metabolic network based on the available enzyme annotation data. Subsequent rounds of simulation and refinement help resolve errors and fill gaps in otherwise incomplete networks [7].

Reactions that were added during subsequent rounds of refinement are flagged in Table A in S1 Tables (ADDED_DURING_REFINMENTS) to distinguish them from the initial set of reactions that were already included in the reconstruction prior to any evaluation or validation steps. Reactions that were removed or added during the refinement steps were enumerated since they are errors that should have been captured during the reconstruction process itself and not to improve model predictions. The rationale for changing these is detailed as follows.

1. Dihydroneopterin aldolase (DHNA) was removed since the corresponding gene is ostensibly missing from Plasmodium genome sequences, and this was a major obstacle to providing a complete description of the folate biosynthesis system. Recent findings discovered an alternative pathway mediated by an unusual variant of 6-pyruvoyltetrahydropterin synthase (PTPS), an enzyme normally involved in BH4 synthesis in other organisms, including humans and which was already in the initial reconstruction version [8].
2. Transport of acetyl-CoA out of the apicoplast (via an unknown transport mechanism) is highly unlikely [9]. Accordingly, the acetyl CoA apicoplast transporter was removed and a sink reaction was added for accoa[h] to retain flux in the apicoplast version of pyruvate dehydrogenase. Also, Cobbold et al. [9] stated that there is likely no acetyl CoA transporter between apicoplast and cytosol. In addition, MPMP maps were in line with [9], so the transporter reaction of acetyl CoA between apicoplast and cytosol was removed.
3. 2-oxoglutarate dehydrogenase reaction was associated with 2-oxoglutarate dehydrogenase E1 component (KDH), dihydrolipoyl dehydrogenase, mitochondrial (LPD1) and dihydrolipoyllysine-residue succinyltransferase component of 2-oxoglutarate dehydrogenase complex, i.e. (PF3D7_0820700 AND PF3D7_1232200 AND PF3D7_1320800). While the literature used to construct the gene rule points to other isozymes that could be involved in catalyzing the KDH reaction, this error was only captured through the gene-essentiality predictions where KDH should be dispensable while the initial model version predicted KDH to be essential for growth (its deletion caused a 100% growth reduction). The *i*TH366 model [10] also had the GPR rule with the correct isozyme 'OR' logic rather than 'AND'. Given that, we corrected the gene rule for this particular reaction and used it as is in the subsequent evaluation and validation steps.
4. Exchange reactions for putrescine, spermidine [11], ornithine [12] and cholesterol [13] were added to the reconstruction. Initially, exchange reactions were incorporated based on *in vitro* growth media composition (RPMI1640 + Hemoglobin + Hypoxanthine). However, since reconstructions represent knowledge bases that should contain all reactions and pathways, uptaken and secreted metabolites should account for all possible growth conditions. Hence, even though these metabolites are not present in standard growth conditions, exchange reactions for these metabolites were added to the generic *i*AM-

Pf480 version. Upon simulating *in vitro* growth conditions, lower bounds for these metabolites were set to zero to mimic the lack of the metabolites in the medium (see validation of *i*AM-Pf480 predicted glycolytic flux rates).

5. An exchange reaction was added for N-acetyl glucosamine to allow (GlcNAc) uptake used for induction of gametocytogenesis[14]. Uptake of GlcNAc was allowed only in the gametocyte stage models (see reconstruction of life cycle stage specific models of *P. falciparum*). Subsequently, N-acetylglucosamine kinase (ACGAMK) and N-Acetyl-D-glucosamine transport via diffusion (ACGAtex) reactions were added to link GlcNAc to the metabolic network.
6. Dihydroxyacetone phosphate transport via triose-phosphate translocator (DHAPThr) removed, Glucose-6-phosphate isomerase (G6PI) was made irreversible to break the loop reaction cycles with triose-phosphate isomerase (TPI) and glucose-6-phosphate isomerase (PGI), respectively.

Validation of *i*AM-Pf480 predicted glycolytic flux rates.

In constraint-based modeling, a solution space is defined to be the one that contains all possible metabolic phenotypes after adding a series of known biologically-relevant governing constraints [15]. Assuming the constraints are accurate, the true steady state flux through the network should be within the *in silico* solution space. The range and distribution of reaction fluxes within these solution spaces are dependent on the constraints, such as reaction thermodynamics, metabolite uptake rates, etc. [15] In this study, we tested whether *i*AM-Pf480-predicted flux rates match previously published kinetic flux data (rapid stable-isotope labeling) of glycolysis in wild-type (WT) and pyruvate dehydrogenase (PDH) deficient *P. falciparum* parasites cultured *in vitro* [9]. Hence, the generic *i*AM-Pf480 model was allowed to uptake metabolites available in standard *in vitro* growth conditions (Table D in S1 Tables). Uptake rates for glucose and hypoxanthine were obtained from literature [16,17]. Uptake rates of other metabolites that are available in standard *in vitro* growth conditions were set to -1. In order to simulate pyruvate dehydrogenase (PDH) knock-out (KO), the apicoplast version of PDH (PDH: PF3D7_1124500) was deleted *in silico* and growth was simulated using identical medium conditions to those used for the wild-type (WT) model (Tables E-F in S1 Tables).

Since properly constrained reactions do not demonstrate uniform distributions of feasible steady-state fluxes, the range and distribution of feasible metabolic flux for each reaction is determined by using Markov Chain Monte Carlo (MCMC) sampling. To do this, a large number of feasible sets of metabolic fluxes are randomly moved within the solution space until they are well mixed, thereby sampling the entire solution space. This sampling process yields a distribution of feasible steady-state fluxes for each reaction [15,18]. Simulations were run and averaged sampled predicted flux distributions for the glycolytic reactions were compared to the experimentally measured flux rates (Fig. 2c-d). There was significant agreement for the predicted flux rates under the tested growth conditions (both in the WT and KO models).

Performance evaluation and validation of *i*AM-Pf480 gene essentiality predictions

We compiled a curated list of experimentally-validated gene knock-outs (n = 21, Table B in S1 Tables) and phenotype resulting from targeted inhibition of enzymatic activities with drugs (n = 59, Table C in S1 Tables) in *P. falciparum,* based on our recently published list of targeted chemical compounds in MPMP [19]. However, since the aim of the current analysis is to evaluate the accuracy of *i*AM-Pf480 in reproducing experimental data from single gene deletion and drug inhibition studies, our list was restricted to data for which compelling evidence does exist i.e., *in silico* or suggested essentiality predictions were not included, only targets whose knock-out or inhibition was done in the context of a lab experiment (*in vitro* for falciparum, *in vivo* or *in vitro* for rodent parasite) were used to assess the performance of the model. The compiled list was manually curated to obtain a final set of high quality experimentally-validated targets whose inhibition in *Plasmodium falciparum* or a rodent malaria caused significant reduction in parasitemia, was lethal, or had no effect on growth. Whenever conflicting reports existed about the essentiality of the target in literature, this target was not used for assessing accuracy. Model-predicted growth reduction of less than 10% was considered non-essential. (see Tables B-C in S1 Tables for a full list of reactions/genes used to evaluate *i*AM-Pf480 performance along with justification for inclusion into the performance evaluation list).

We estimated the performance of *i*AM-Pf480 under open constraints (Tables B-C in S1 Tables) as well as under standard *in vitro* growth conditions (iAM-Pf480 *in vitro* Tables B-C in S1 Tables) (see validation of *i*AM-Pf480-predicted glycolytic flux rates for details). The models were validated by true-positive and true-negative (TN) results that highlight cases where the models were in agreement with experimental results. In contrast, false-positive and false-negative cases indicate potential errors or gaps in the models (missing knowledge) or missing context-specific information such as transcriptional regulation [20]. Furthermore, in order to estimate the significance of the accuracy of the model's predictions, we computed Fisher exact test as well as Mathew correlation coefficient (MCC) for the single gene deletion experiments (Fig. 2). Results showed that model predictions for single gene deletion experiments were significantly accurate with a *p*-value of 6.88e-05 (Fig. 1b). A *p*-value could not be computed for the accuracy of model predictions for the drug inhibition experiments since the total number of TN was zero. The results of gene essentiality analysis are summarized in Fig. 1b.

Comparison of *i*AM-Pf480 to previously published *P. falciparum* models

Comparison to *i*TH366[10]

Our initial assessment of *i*TH366[21] raised some concerns that we felt necessitated a new reconstruction to be generated. In particular the failure of mass conservation in *i*TH366; we found that *i*TH366 was able to produce almost all amino acids even after all uptake reactions were shut off namely: [ala-L, arg-L, asn-L, asp-L, cys-L, gln-L, glu-L, gly-L, his-L, Leu-L, Lys-L, met-L, phe-L, pro-L, ser-L, thr-L, trp-L, tyr-L, val-L] in addition to multiple duplicated reactions (28 that were initially identified, e.g. NTD8pp is the same as NTD8). Given these issues we determined it would be more efficient to generate a new reconstruction within standard quality

control checks procedure and pipeline rather than attempt to correct errors and update the content of *i*TH366.

Performance assessment of our model, *i*AM-Pf480, using experimentally validated single gene deletion experiments and drug inhibitors showed that *i*AM-Pf480 predictions were more accurate than *i*TH366 under the same conditions (unconstrained uptake rates) (Fig. A). Particularly, *i*AM-Pf480 accuracy was 90% while that of *i*TH366 was 81% using experimentally validated single gene deletion experiments (Fig. A.a). Similarly, *i*AM-Pf480 accuracy was 51% while that of *i*TH366 was 39% using the experimentally validated drug inhibitors (Fig. A.c). Further, when simulating growth under standard *in vitro* growth conditions (Tables B-C in S1 Tables), *i*AM-Pf480 accuracy was 95% and 71% for the single gene deletions and drug inhibitors, respectively.

a

	*i*AM-Pf480	*i*TH366
**Included genes**	480	366
**Reactions**	1082	1001
**Gene associated (metabolic/transport)**	738 (68%)	657 (66%)
**No gene association (metabolic)**	76 (7%)	72 (7%)
**No gene association (transport)**	159 (15%)	161 (16%)
**Exchange**	92	108
**Demand/Sink**	16	2
**Blocked**	223 (21%)	334 (33%)
**Metabolites**		
**Unique metabolites**	617	616
**Cytoplasmic**	531	537
**Apicoplast**	109	135
**Golgi**	45	-
**Mitochondria**	82	83
**Endoplasmic reticulum**	26	-
**Lysosome**	9	-
**Extracellular**	107	161

b

Experimental

Prediction	essential	non-essential
essential	6	0
non-essential	2	13

*i*AM-Pf480 (overall accuracy = 90%)
MCC = 0.81

Prediction	essential	non-essential
essential	5	1
non-essential	3	12

*i*TH366 (overall accuracy = 81%)
MCC = 0.56

c

Experimental

Prediction	essential	non-essential
essential	28	1
non-essential	30	--

*i*AM-Pf480 (overall accuracy = 47%)

Prediction	essential	non-essential
essential	23	1
non-essential	35	--

*i*TH366 (overall accuracy = 39%)

Figure A. Comparison between *i*AM-PF480 and the previously published *P. falciparum* model *i*TH366 [9] a) *i*AM-Pf480 features in comparison to *i*TH366. Performance evaluation of *i*AM-Pf480 *vs*. *i*TH366 under open constraints using our manually curated list of b) single gene deletion experiments and c) drug targets.

<u>Comparison to *i*Pfa[22]</u>

Gene content comparison. *i*Pfa[22] has 318 genes (corresponding to 325 transcripts) and 670 metabolic reactions. At the gene content level, *i*AM-Pf480 and *i*pfa have in common 234 genes. *i*AM-Pf480 has 246 genes that are not in *i*Pfa while *i*Pfa has 85 genes that are not included in *i*AM-Pf480: 43 (50%) of those missing from *i*AM-Pf480 are tRNA ligases, and the rest are mostly of putative or non-metabolic function (e.g. myosin). We already provide bibliomic evidence for inclusion of all gene-reaction association for *i*AM-Pf480 in Table A in S1 Text. Nearly 13% of metabolic reactions in *i*pfa doesn't have gene associations (orphan). Although the size of *i*AM-Pf480 network is larger (1083 reactions), the number of orphan reactions is only 7% (Fig. 2a).

Compartmentalization. *i*AM-Pf480 accounts for detailed compartmentalization of enzymes in *P. falciparum*. For example, the hemoglobin digestion reactions which are supposed to occur in the digestive vacuole are included in *i*Pfa but are localized to the cytosol, the authors indicated

that they have merged compartments which included few enzymes (e.g. digestive vacuole and Golgi apparatus) with the cytosolic compartment.

Functional comparison. In the supplementary methods[22], the authors of the *i*Pfa model have indicated that they integrated experimentally measured uptake rates for glucose, and isoleucine and constrained lactate secretion rate. The model as downloaded from the LCSB database has constraints only for isoleucine. The constraints are imposed on the transporter reaction (R_T_c_to_e_C00407) between the extracellular and cytosolic compartments of the model rather than on the uptake reaction (EXC_BOTH_C00407_e). We applied the glucose uptake and lactate secretion rates according to the supplementary material (0.62 and 0.76mmol/h-gDW) and the predicted growth rate matched that reported by the authors (0.16 h-1).

We were able to reproduce the results of table SVI from the *i*Pfa model paper and hence, used the same evaluation criteria to compare *i*Pfa and *i*TH366 against our model (Table L).

Table L| Performance evaluation of *i*AM-Pf480, *i*Pfa[22] and *i*TH366[10] (reproduced from the supplementary material of the *i*Pfa model paper[22])

	*i*TH366	*i*Pfa	*i*AM-Pf480 (without in vitro growth constraints)
**Number of *ad hoc* reaction directionality**	590 (59%)	185 (14%)	155 (14%)*
**Number of blocked reactions**	334 (33%)	312** (24%)	222 (21%)
**Number of essential genes**	60*** (16%)	55 (17%)	106 (22%)

*assuming '*ad hoc* directionality' means no evidence to support a specific reaction directionality, hence the reaction was assumed to be reversible. The set of 155 reactions is composed of reversible reactions for which there is no 'PMID' or 'MPMP' entry in Table A in S1 Tables from our paper.
**the supplementary material from the *i*Pfa model reads 313 (24%)
***the supplementary material from the *i*Pfa model reads 57 (16%)

The number of blocked reactions in *i*AM-Pf480 was much less (21%) than *i*Pfa[22] (24%). Worth mentioning, when testing the number of essential genes in iPfa, we were able to reproduce the results of having 55 genes as essential, however, the deletion of all 55 genes totally inhibited growth (growth reduction percentage = 100%), while in case if *i*AM-Pf480 the cutoff threshold for considering a gene to be essential was a growth reduction of more than 10%, i.e. the effect of deletion of any gene in *i*Pfa was either a 0% or 100% reduction in growth.

Performance assessment using our curated set of experimentally validated targets.
Using our manually compiled single gene deletion experimentally validated set (n = 21) (Table B in S1 Tables).

Two genes were not in *i*Pfa: ribonucleotide reductase small subunit (PF3D7_1015800) and adenylyl cyclase alpha (PF3D7_1404600) although experimental evidence exists for the essentiality of the former (PMID: 8265664) and non-essentiality of the later (PMID: 18389080). Both genes are also present in *i*TH366. The overall prediction accuracy using *i*Pfa was 38% (cf. 95% for *i*AM-Pf480 (under standard *in vitro* growth conditions) and 81% for *i*TH366)

Using our drug targets list (n = 59) (Table C in S1 Tables).

When using our manually curated list of targets, for which drug inhibitors have been experimentally tested for activity in *P. falciparum* to evaluate *i*Pfa performance:

5 drug targets couldn't be tested since the corresponding reaction(s) were missing from *i*Pfa. Overall, *i*Pfa could correctly predict the essentiality of 13 drug target (22%) out of the 59 tested targets (cf. 71% and 39% for *i*AM-Pf480 under standard *in vitro* conditions and *i*TH366, respectively)

Performance assessment using *i*Pfa-predicted essential genes. *i*AM-Pf480 was able to predict 29/55 (52%) of the essential genes predicted by *i*Pfa without accounting for thermodynamic constraints. This percentage increased to 54% (30/55) when using *i*AM-Pf480 while simulating *in vitro* growth conditions. Interestingly, palmitoyl-CoA:L-serine C-palmitoyltransferase and ATP:UMP phosphotransferase were predicted as essentiali by *i*AM-Pf480 whereas *i*Pfa was able to predict their essentiality only after applying the TFA framework and MS data. Its possible that the discrepancy between the two models is due to the difference evaluation criterion with regards to affecting growth in case of *i*AM-Pf480 *vs*. affecting metabolic tasks in *i*Pfa.

Comparison to *i*PfaI17[23]

In comparing the single gene deletion and drug inhibition data results, it was found that *i*PfaI17 correctly predicted 86% and 46%, respectively (*i*AM-Pf480 predicted 90% and 47%). *i*PfaI17 and *i*AM-Pf480 share 367 genes. *i*PfaI17 is noted to be a larger model with 109 genes that are not in *i*AM-Pf480, however 81% of those involve tRNA metabolism (which are in turn included in the biomass), but don't have any direct role in biochemical transformations on the network.

***P. falciparum* life cycle stage-specific model building and validation procedures.**

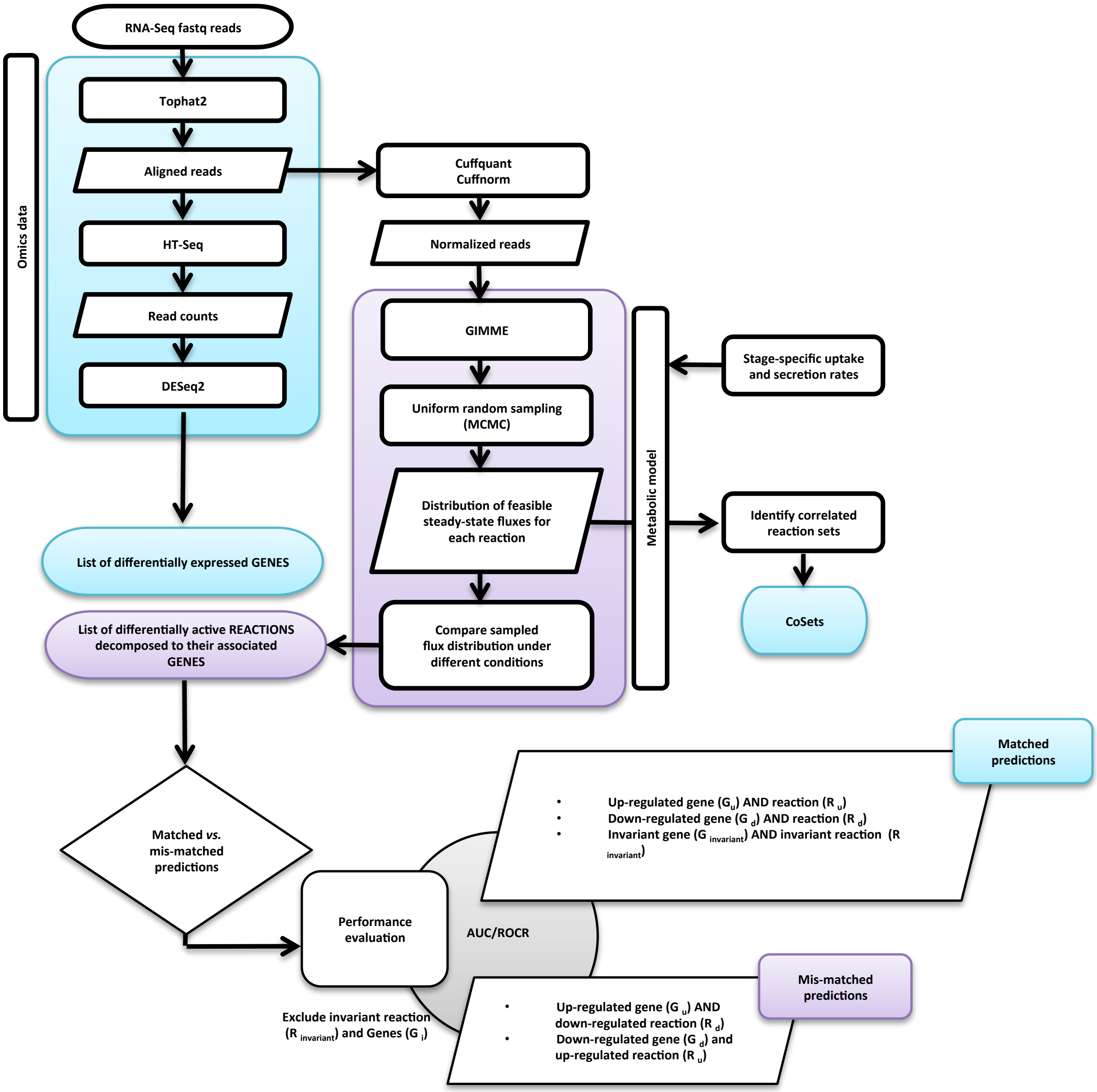

Figure B. *P. falciparum* life cycle stage-specific model reconstruction and performance evaluation workflow. Stage- and species-specific models were generated first by using stage-specific uptake and secretion rates (see below) as well as using stage-specific transcripomic data. Log_2 normalized RNA-Seq reads (RPKMs) were mapped to *i*AM-Pf480. RPKMs were obtained using Cuffquant/Cuffnorm pipeline[24]. GIMME algorithm[25] was used for the integration step. Differential gene expression analysis was carried out between every two stages from the same species using DESeq2[26] and the raw read counts as computed by HTSeq [27]. The lists of significantly differentially expressed genes were compared against the list of genes associated to reactions whose predicted flux rates are significantly changed between the same two stages by computing the area under the ROC curve (AUROC) (Fig. 3b-c) using the ROCR package[28].

The WT *P. falciparum* model (see validation of predicted glycolytic flux rates and Table D in S1 Tables) was used as the template model to generate the life-cycle stage specific models. In addition the following uptake/secretion rates were imposed on the stage-specific models:

- For the trophozoite and schizont models, lactate secretion was constrained to 93% of the glucose internalized by the asexual stages[29]. The growth rate was constrained to a minimum of 0.045 mmol/gDW/h corresponding to 15h doubling time where the range of doubling times reported for asexual stages replication was between 7-9h[30].
- For the early gametocyte (GII) stage model, lactate secretion was constrained to 80% of the glucose uptake[29] and N-acetylglucosamine (GlcNAc) uptake was allowed[14]. The constrain on the lower bound of the growth rate was relaxed since its expected that the proliferation rate of the gametocyte stages is less than that of the asexual stages.
- For the late gametocyte (GV) stage model, lactate secretion was constrained to 80% of the glucose uptake[14] and N-acetylglucosamine (GlcNAc) uptake was allowed[14]. No growth was allowed and the objective function was set to maximize cytosolic ATP production[31].
- For the ookinete stage model, glucose uptake was reduced to 10% of the uptake rate of the asexual stages to mimic a glucose-rare environment[29].

Following, applying the stage-specific uptake and secretion rates, stage-specific transcriptomic data was used to further constrain the stage-specific models following the workflow depicted in Fig. B. *P. falciparum* 3D7 life cycle stage-specific RNA-Seq data was downloaded from SRA archive (SRP009370)[32] SRA files were converted to fastq files using the sra-toolkit[33]. Tophat2[34] was used for the alignment (--library-type fr-unstranded) libraries. PICARD (http://broadinstitute.github.io/picard/) and samtools[35] were used for processing the aligned reads and HTSeq[27] was used to produce read counts (--stranded=no).

Performance evaluation of stage-specific models against differential gene expression (DEG) analysis results.

Previous studies have demonstrated that while flux does not always correlate with gene expression levels, changes in flux often correlate with changes in gene expression levels or the abundances of active enzymes[15,36]. Thus, we wanted to assess the consistency between experimental differential gene expression (DEG) and changes in model-predicted flux rates. Stage-specific model predictions were compared against DEG following the workflow previously introduced in [15] and outlined in Fig. B. Briefly, differential gene expression analysis was carried out between every two stages from the same species and the lists of significantly differentially expressed genes (DEG) (FDR < 0.05 and (> 75th or < 25th percentile of the log2 fold change in expression)) were later used for evaluation of stage- and species-specific models' predictions. For each reaction, a distribution of feasible steady-state flux values was acquired from the uniformly sampled points using MCMC (see validation of *i*AM-Pf480 predicted glycolytic flux rates). The list of reactions that show significantly different (FDR < 0.05 and (> 75th or < 25th percentile of the log2 fold change in reaction fluxes)) distribution of possible fluxes for the two stages is returned, along with the direction of the change in magnitude. All significantly changed

fluxes are then decomposed into their associated genes using the gene-protein-reaction associations. Through this, lists of genes that are predicted to be up-regulated or down-regulated depending on their associated reactions are obtained and are compared to DEG lists (Fig. B and Table M). Genes that are associated both with reactions that increase and other reactions that decrease as well as loop reactions were removed from the analysis.

Table M| Performance evaluation of the life-cycle stage specific models of *P. falciparum*

P. falciparum	Schizont vs. T	GV vs. T	Ook vs. T
**AUC**	0.71	0.79	0.67
**MCC**	0.87	0.32	0.5
**Size of model 1**	891	891	891
**Size of model 2**	920	787	891
**Rxns unique to model 1**	36	145	61
**#Rxns unique to model 2**	65	41	61
**#Upreg rxns**	99	207	101
**#Upreg GPRs**	37	79	53
**#Downreg rxns**	49	218	192
**#Downreg GPRs**	31	151	102
**#Upreg genes rnaseq**	55	69	63
**#Downreg genes rnaseq**	58	63	68
**#Consistent predictions**	32	39	51
**#Inconsistent predictions**	2	20	7

Co-Sets predict a stage-dependent fate of glucose-6-phosphate in *P. falciparum*

Interestingly, in the late gametocyte stage (GV), the non-oxidative branch was correlated with inositol metabolism (Figure. 4). Glucose-6-phosphate (G6P) is a substrate of myo-Inositol 3-phosphate synthase (MI3Ps), which produces myo-inositol 3-phosphate in the first step of inositol metabolism[37]. G6P is also an intermediate metabolite of the glycolysis pathway (Figure. 4). Therefore, it's possible that inositol metabolism and glycolysis compete for G6P[37]. It's well established that the asexual stages are dependent on glycolysis to support their rapid proliferation[38] while the mature sexual stages are thought to be metabolically quiescent [31,38]. A possible scenario then is that in the asexual stages, because glycolysis is more dominant, more G6P is directed to the lower branch of glycolysis for ATP generation through aerobic glycolysis. In contrast, during the gametocyte stage, more G6P is diverted into inositol metabolism, since glycolysis is less utilized in this stage[31]. Inositol metabolites have been shown to be protective against abiotic stress in plants[37]. In malaria, environmental stressors, such as high host parasitaemia or drug treatment, are among the established cues for sexual commitment[39], so its possible that more inositol is needed to protect the gametocytes against the various stressors. Interestingly, phosphatidylinositol 4-kinase (PI4K) has been shown to be an essential and druggable target that blocks development at multiple stages of the Plasmodium lifecycle, underscoring the importance of inositol metabolism across several life cycle stages of malaria[40].

Interestingly, a recent study[41] reports that the asexual stages are dependent on de novo synthesis of myo-inositol through MI3PS but its essentiality in sexual stages was never tested before. Other genes that were essential only in the late gametocyte stage, IMP-specific 5'-nucleotidase (PF3D7_1206100) and phosphoinositide-specific phospholipase C (PF3D7_1013500).

Additionally there are a few other observations that follow from assessment of the co-sets (Table G in S1 Tables). Isoleucine is the only amino acid correlated with growth rate in the proliferating stages since it is absent from adult human hemoglobin[42]. Lysine degradation correlated with lipecolic acid secretion in all stages[43]. Other amino acids were correlated with hemoglobin degradation and hemozoin formation (Table G in S1 Tables). Furthermore, the trophozoite model predicted flux rates confirmed the hypothesized role of fumarate hydratase (FH) in purine salvage where FH and malate dehydrogenase (MDH, MDH6, MDH7m) were correlated with adenylosuccinate lyase (ADSL), aspartate transaminase (ASPTA) and adenylosuccinate synthetase (ADSS). FH was one of two enzymes of TCA that couldn't be knocked out during IDC, although all other 6 enzymes of TCA cycle were knocked out without affecting asexual growth suggesting a role of FH in non-TCA related function[44,38]. The stage-specific model predictions showed that ADSL and ADSS, which are involved in the purine salvage pathway[38], are correlated with FH. Hence, confirming the non-TCA role of FH in nucleotide metabolism in the asexual stages of *P. falciparum*.

Species-specific model building procedure

<u>Reconstruction of species-specific models.</u>

Although automated reconstruction methods exist and are powerful tools for creating draft metabolic reconstructions for a wide range of species, it has been shown that starting from the highly curated content of a closely related organism produces a more accurate model than the currently available automated methods [20]. Fig. C outlines the details of the workflow followed in this study to generate the malaria multi-species models. Genome-scale metabolic models were reconstructed for 4 Plasmodium species (other than *i*AM-Pf480 for *P. falciparum*) that infect humans, non-human primates and rodents. These are *P. vivax 'P.viv', P. knowlesi 'P.kno', P. cynomolgi 'P.cyn', and P. berghei 'P.ber'*).

Starting from genome annotation of a target species (e.g. *P. berghei*), two approaches were followed to decide which reactions to add or remove from the original generic *P. falciparum* network. The transform by orthology tool from plasmodb.org was used for generating the reciprocal orthologs across all four species for metabolic genes of the *i*AM-Pf480. Using this tool, three groups of genes were generated: 1) Orthologous genes that exist in *P. falciparum* and target species, 2) Genes that are unique to *P. falciparum* (i.e., with an orthologs count in target species = 0) whose corresponding reactions were removed, and 3) genes that are unique to target species (i.e., orthologs count in *P. falciparum* = 0) whose corresponding reactions were added to the network following the same steps in the reconstruction of the generic *P. falciparum* model.

When genes were annotated as ‘hypothetical with unknown function’, a 2-way BLAST was carried out and according to the percentage of sequence similarity (cutoff 50%) between *P. falciparum* and the target species’ genes as well as function prediction using I-TASSER (RMSD =< 5) [45-47] a decision was made on whether to add or remove the corresponding reaction to the target species genome-scale metabolic model under reconstruction. Further, a data set composed of 101 one-to-one orthologs across 26 alveolate and stramenopile species [48] was queried for orthologous groups discrepancies between Plasmodium species (*P.fal, P.kno, P.viv, P.ber*). All genome annotations were downloaded from [PlasmoDB 24](). In addition, [KEGG-REST]() was checked for all plasmodium species available through KEGG REST (pfa, pyo, pvx, pkn, pcb, pber and pcy).

Orthologous groups were assigned to each gene in each species-specific model (Fig. C) where 422 orthologous groups were common across all 5 species. A few genes are seemingly functionally similar, so they have been assigned to the same reaction during the reconstruction process. However, they belong to different orthologous groups across the different species: 1) Copper-transporting ATPase (CuTP) for *P.fal*, *P.kno* and *P.ber*: OG5_126855 while *P.viv* and *P.cyn* copper-transporting ATPase (CuTP) belong to a different orthologous group: OG5_168155. 2). Amino acid transporter, putative for *P.kno* belongs to OG5_188855, *P.fal*, *P.viv* and *P.cyn* belong to OG5_129703 and *P.ber* belong to OG5_126804. 3) N-acetylglucosaminylphosphatidylinositol deacetylase, putative for *P.kno* belong to a different orthologous group (OG5_158530) while other species N-acetylglucosaminylphosphatidylinositol deacetylase belong to OG5_128077.

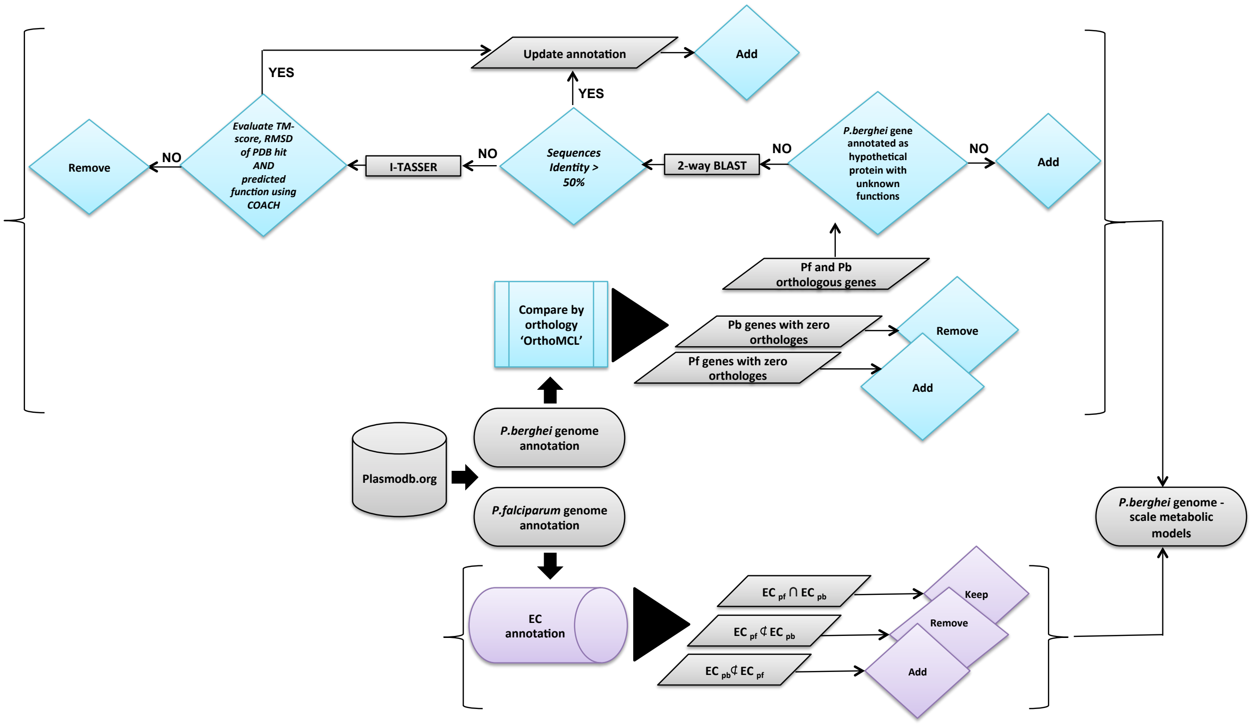

Figure C. Multi-species reconstruction workflow. Details are explained in the text.

The *P. falciparum* reconstruction was the largest among the five species, with 480 genes associated with 1083 reactions. *P. berghei* was the smallest reconstruction with 448 genes associated with 1067 reactions (Table N); these findings are consistent with differences in genome size of rodent (~18-20MB) and non-rodent malaria (~23-24MB), respectively

Table N| The size of each species-specific reconstruction in terms of reaction (GEM reactions) and gene content (GEM genes) compared to the genome size (Size) and number of annotated genes (*total number of annotated genes obtained from Plasmodb.org Release 26, 14th of October*

	Size (Mbp)	Genes	GEM genes	GEM reactions
P. facliparum	23.33	5777	480	1083
P.vivax	27.01	5626	461	1078
P.knowlesi	24.40	5483	459	1079
P.cynomolgi	26.18	5776	455	1074
P.berghei	18.78	5254	448	1067

Curation notes for specific enzymes that differed across the species.

Carbonic anhydrase (CA).

The carbonic anhydrase gene is only present in *P.fal* and *P.ber* while the gene is not found in *P.viv*, *P.cyn* nor *P.kno*. Inhibition of CA-associated reactions caused 100% reduction of growth since it generates bicarbonate for Carbamoyl-phosphate synthase. Further, there is ample evidence for spontaneous occurrence of the reaction albeit at lower rate. Hence, the reaction was kept in *P.viv*, *P.cyn* and *P.kno* but with no gene association.

Sepiapterin reductase.

Sepiapterin reductase (SPR) and 6-pyruvoyltetrahydropterin synthase (PTHPS) were unable to carry flux in the rodent and *P.cyn* models while being active in the human and *P.kno* models. The PTHPS gene encodes an unusual orthologue of 6-pyruvoyltetrahydropterin synthase (PTPS), which in mammals is part of the tetrahydrobiopterin (BH4) biosynthetic pathway. *P. fal* has a pterin-4a-carbinomaline dehydrates (PCD) activity (reaction producing the cofactor BH4; PF3D7_1108300). This enzyme does not have any orthologs in the rodent malaria, nor in *P.cyn*. In general, the possible usage of BH4 is for amino acid hydroxylases, alkyl mono glycerol ether or NOS activity. AA hydroxylases and alkyl mono glycerol oxygenases are not detected in malaria. Since *P. fal* has NOS activity whilst being absent from rodent species, a possible hypothesis is that human malaria retains BH4 metabolism for NOS activity while rodent malaria does not.

Nitric oxide synthase.

In the reconstruction, we accounted for reactions whose gene association have not been identified nor characterized but for which there is evidence for their occurrence in Plasmodium. Nitric oxide synthase (NOS) activity was detectable in *P. fal* [49] but in none of the rodent malaria species. This is consistence with the absence of pterin-4a-carbinolamine activity in rodent malaria [50,51]. Also, studies with iNOS−/− knockout mice found parasitemia levels similar to that of control animals [52]. PF3D7_1459100 (annotated as GTP-binding protein in Plasmodb) is associated to the orthologous group OG5_128338 whose members possess a nitric-oxide synthase activity (1.14.13.39), We have not associated PF3D7_1459100 to the corresponding reaction in our reconstruction but we have accounted for the nitric oxide synthase activity. Recently, Cobbold et al. [53] reported that PF3D7_0923200 is a putative NOS. Although the reaction is already included in our non-rodent models, we have not included the associated gene as there is no demonstration of nitric oxide synthase activity. Also, NOS reaction is only included in the non-rodent models assuming that BH4 metabolism is linked to NOS activity.

COX1 and COX3.
No orthologues were detected for COX1 and COX3 in *P. cyn* nor for cytochrome b (CYTB) (mal_mito_1, mal_mito_2 and mal_mito_3 orthologous) in Plasmodb.org. Initially, the reaction CYOOm2 was removed from *P.cyn* reconstruction since the gene association rule for this reaction contains 'AND' logic. However, we found several studies using CYTB, COX1 or COX3 to construct phylogenetic trees for plasmodium species where *P. cyn* was among the species that possess all three genes, and hence we added this reaction back to *P.cyn* reconstruction and removed it from the pan not core reactions. In fact, these genes were annotated for *P. cyn* in genbank (accession numbers: AF069616, AB444126, AB471873). The annotation of these genes should be updated in Plasmodb.org.

Quinone oxidoreductase.
Only one gene associated to one reaction, NADPH: quinone oxidoreductase (PBANKA_0708600) was in the pan Plasmodium metabolic content but not in *P. fal* reconstruction. In fact, this gene was restricted to the rodent parasites (*P. chabaudi, P. berghei and P. yoelii*) while having no ortholog in the non-rodent malaria (*P. falciparum, P. vivax, P. cynomolgi and P. knowlesi*) (Table I in S1 Tables). Nevertheless, the major contributor to the mitochondrion electron transport chain, type II NADH:ubiquinone oxidoreductase (NDH2) [54,55], was found in all species analyzed in this study.

Host-specific hemoglobin composition

The malaria parasite uses host erythrocyte hemoglobin as a major nutrient source[56]. To further contextualize the malaria species-specific models, we accounted for the differences host hemoglobin composition as retrieved from uniprot (Table O). The stoichiometric coefficients in the hemoglobin digestion equations, as well the molecular formulae for hemoglobin, alpha and beta chains were accounted for in each species-specific model.

Table O| Host-specific hemoglobin composition

	alpha-chain		beta-chain	
	Length	Uniprot ID	Length	Uniprot ID
Homo sapiens (pfal, pviv)	142	P69905	147	P68871
Macaca_mulatta (pcyn)	142	P63108	146	P02026
Macaca_fascicularis (pkno)	141	P21767	147	P68223
Rattus_norvegicus (pber)	142	P01946	147	P02091

Validation of the species-specific reconstruction workflow

For validation, we assessed the performance of the rodent-infecting malaria model, *i*AM-Pb448 (*P. berghei*) predictions for gene essentiality by comparing *i*AM-Pber448 predictions to single gene deletion experiments (phenotypes) reported in the Rodent Malaria genetically modified Parasites DataBase (RMgmDB) (Table J in S1 Tables). When simulating standard *in vitro* growth conditions (similar to *P. falciparum*), *i*AM-Pber448 correctly predicted 80% of the experimentally tested phenotypes (Table J in S1 Tables and Fig. D). To our knowledge, this is the first rodent-infecting malaria genome-scale metabolic model as well as the first set of non-human malaria infecting species metabolic models.

Experimental

Prediction

	essential	non-essential
essential	10	6
non-essential	10	60

*i*AM-Pb448 (overall accuracy = 81%)
MCC = 0.44

	essential	non-essential
essential	9	6
non-essential	11	60

*i*AM-Pb448 simulating standard growth conditions (overall accuracy = 80%)
MCC = 0.4

Figure D| Performance evaluation iAM-Pb448 against experimentally validated data from RMgmDB

<u>Metabolic Similarities across Plasmodium species</u>

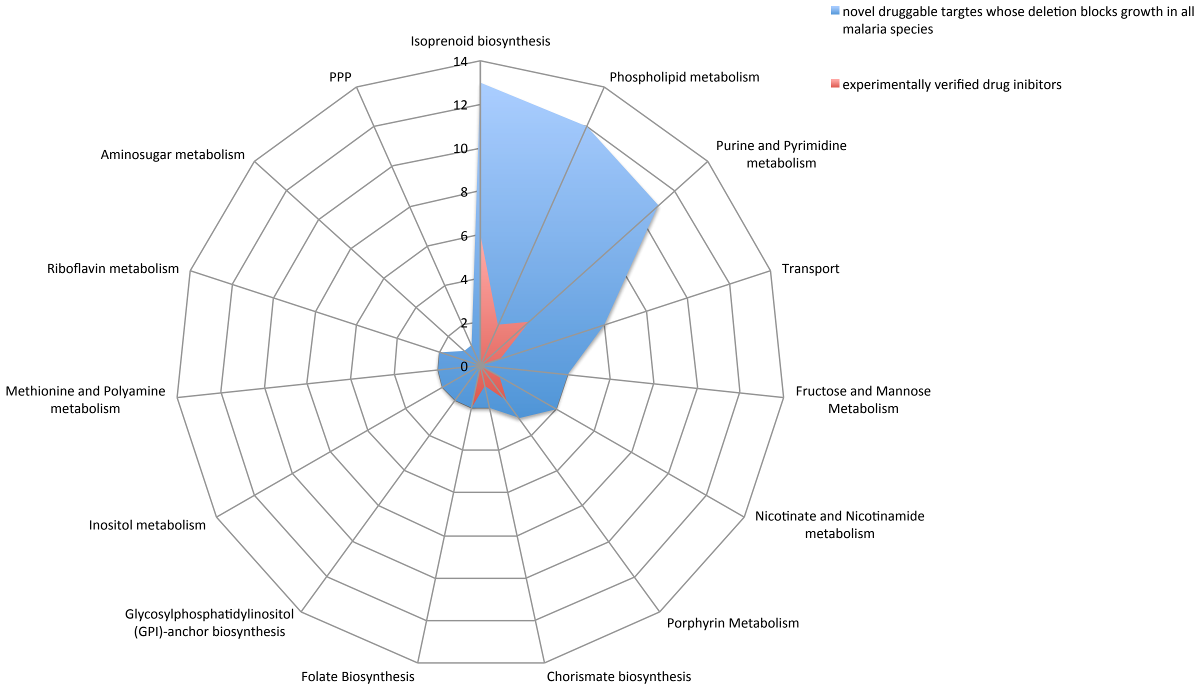

Figure E| Similarities in metabolic capacities of *Plasmodium* species. Druggable targets (n = 67) whose deletion resulted in blocking of growth across the human, non-human primate and rodent malaria infecting species modeled in this study. The proportion of novel targets in each metabolic subsystem is indicated in blue relative to the experimentally validated druggable targets (Table C in S1 Tables) indicated in red.

We discuss three of the growth-related conserved targets that are predicted by the multi-species models to be essential for growth of malaria species: 1) Phosphomannomutase (PMM) is predicted by our models to be essential across all Plasmodium species. PMM is involved in fructose and mannose metabolism where it produces GDP-mannose (GDP-Man), the activated form of mannose. GDP-Man is incorporated in the GPI-anchor of the blood stages of the parasite[57], the major glycosylated molecules on the surface of the Plasmodium parasite essential for primary contact with the host red blood cell[58,59]. Hence, we hypothesize that disruption of PMM would interfere with the pathogenesis of malaria. 2) Mitochondrial cardiolipin synthase (CLS) is another overlooked candidate target predicted by the species-specific models to be essential in all malaria species. Deletion of CLS results in the death of *Trypanosoma brucei*, the causative agent of human African sleeping sickness[60]. In eukaryotes, CLS is required for proper function of key mitochondrial enzymes and proteins involved in oxidative phosphorylation and certain mitochondrial transport systems. In addition, mitochondrial CLS associates with several respiratory complexes[60]. Hence, CLS inhibition is expected to compromise mitochondrial function in life cycle stages where oxidative phosphorylation is more active[38,61]. 3) Glutamine-dependent NAD(+) synthetase was predicted to be equally potent in

blocking growth across all malaria species to nicotinate-nucleotide adenylyltransferase (NMNAT). Elevated NAD+ synthesis driven by the parasite promoted the design of inhibitors against NMNAT[62,10], however, NADS efficiency in reducing malaria growth was not experimentally tested before.

Variations in metabolic capacities between rodent- and human-infecting malaria species

Reactions with the most striking differences in activity across malaria species were between the rodent and non-rodent species, namely: thiamine pyrophosphokinase (TPK), and choline kinase and a member of the holo-acid dehalogenase (HAD) superfamily protein. All three genes were predicted to be essential in *P.berghei* only, while their deletion had no effect on growth in any of the non-rodent species. TPK and CK are discussed in the main text.

In non-rodent malaria models, a holo-acid dehalogenase (HAD) superfamily protein (PF3D7_0303200) and a PAP2-like protein (PF3D7_0805600) catalyze the synthesis of diacyl glycerol (DAG) from phosphatidic acid[63]. No rodent malaria ortholog exists for the PAP2-like protein. Hence, the deletion of the rodent malaria ortholog of the holo-acid dehalogenase (HAD) superfamily protein blocks synthesis of DAG, the precursor for the synthesis of most phospholipids and triacylglycerols, from phosphatidic acid, eventually, inhibiting growth of the rodent model. In a recent study, another closely related HAD (PfHAD1) has been shown to control deoxyxylulose 5-phosphate (DXOP) availability, a key metabolite in the synthesis of isoprenoids. Its also possible that HAD plays a dual role in case of rodent-infecting species where it catalyzes the reaction for DAG generation as well as regulating availability of key metabolites.

Phylogenetic analysis using TPK and CK *vs*. DHODH.

Although phylogenetic trees illustrate how different/similar species are based on sequence similarity, they seldom give mechanistic insights into the functional impact of these differences/similarities. To illustrate the utility of the species-specific models in comparison to phylogenetic analysis, we generated three phylogenetic trees (Fig. F) based on thiamine pyrophosphokinase (TPK), choline kinase (CK) and dihydro-orotate dehydrogenase (DHODH) using pre-built workflow from Phylogeny.fr platform[64,65]. The workflow comprised the following steps. First, sequences were aligned with MUSCLE (v3.8.31) configured for highest accuracy (MUSCLE with default settings). Second, ambiguous regions (i.e. containing gaps and/or poor alignment) were removed with Gblocks (v0.91b) using 10 as the minimum length of a block after gap cleaning. No gap positions were allowed in the final alignment, all segments with contiguous non-conserved positions bigger than 8 were rejected, and the minimum number of sequences for a flank position was 85%. Third, the phylogenetic tree was reconstructed using the neighbor joining method implemented in the BioNJ program, with 1000 bootstraps. Fourth, graphical representation and edition of the phylogenetic tree were performed with TreeDyn (v198.3).

As explained in the main text, both TPK and CK are essential in rodent species but only minimally affect growth in non-rodent plasmodium species (while thiamine uptake is allowed).

DHODH is an essential gene and drug target across both rodent and non-rodent species (Tables B-C in S1 Tables). Interestingly, the three trees were identical topologically although the models predictions supported by literature evidence have demonstrated that TPK and CK are essential only in rodent species while being dispensable in non-rodent ones.

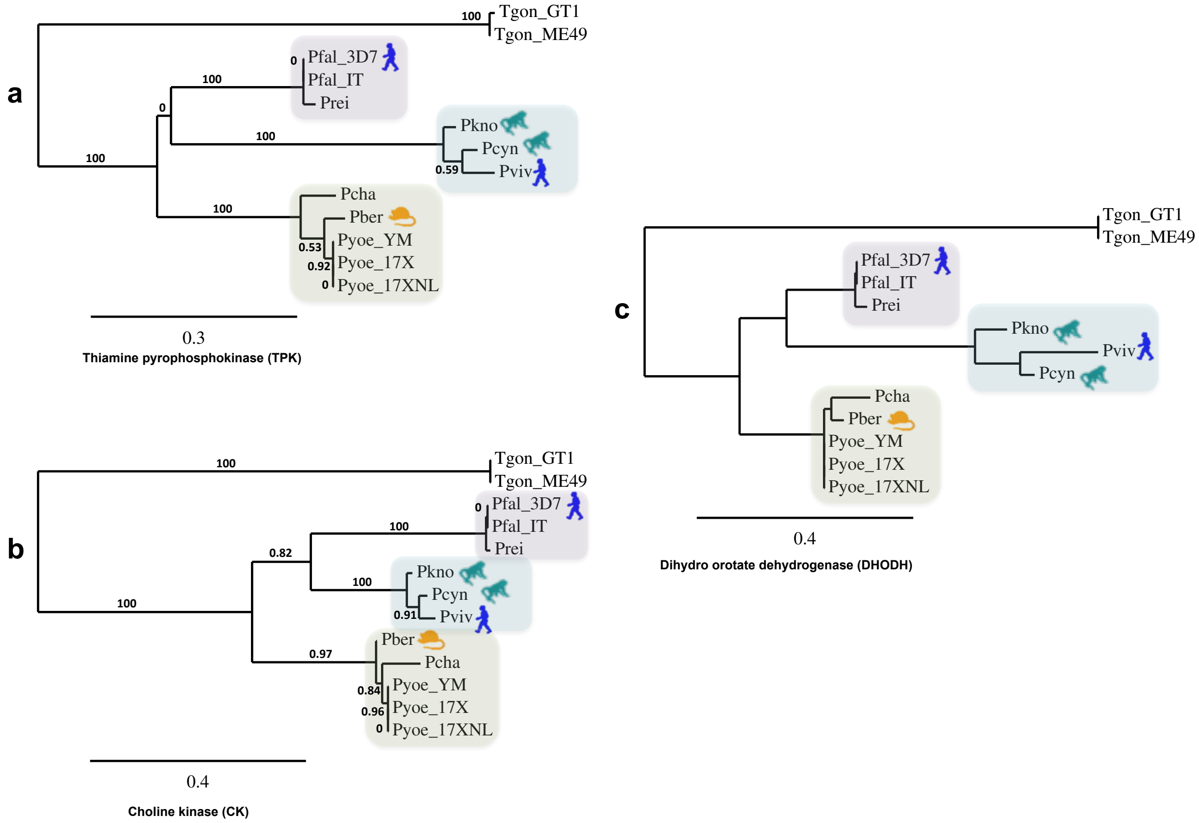

Figure F| Phylogenetic trees using a) TPK, b) CK and c) DHODH. CK vs DHODH phylogenetic trees show identical topology at the clade level. Although within the sub-clades there are some minor differences, the bootstrap values of these differences are Tgon: *Toxoplasma gondii*, Pfal: *Plasmodium falciparum*, Prei: *Plasmodium reichenowi*, Pkno: *Plasmodium knowlesi*, Pcyn: *Plasmodium cynomolgi*, Pber: *Plasmodium berghei*, Pyoe: *Plasmodium yoelii*

Life cycle stage-specific models of *P. berghei*

Following the same procedures that were used to generate the *P. falciparum* stage-specific models, we generated life-cycle stage-specific models of *P. berghei*. The same uptake and secretion rates that were imposed on *i*AM-Pf480 were also imposed on *i*AM-Pb448. Following, previously published *P. berghei* stage-specific transcriptomic data was used to constrain the rodent-stage-specific models. *P. berghei* ANKA life stages transcriptomic data was downloaded from SRA archive (SRA093247)[66] and were processed following the same procedures for *P. falciparum* (see *P. falciparum* life cycle stage-specific model building and validation procedures).

To allow valid comparison between the stage-specific models of *P. falciparum* and *P. berghei*, the raw counts for each species were merged by orthologous group (summation of read counts per gene) before normalization by DESeq2[26] (Fig. B). The sexual stages of both species clustered separately from the asexual stages (Fig. G), confirming that the clustering is not due to batch effect. Also, the distribution of the normalized counts of the raw reads of the different stages from the two species was comparable (Fig. G). Following, log2 normalized RNA-Seq reads (RPKMs) were used to generate the stage-specific *P. berghei* models (Fig. G).

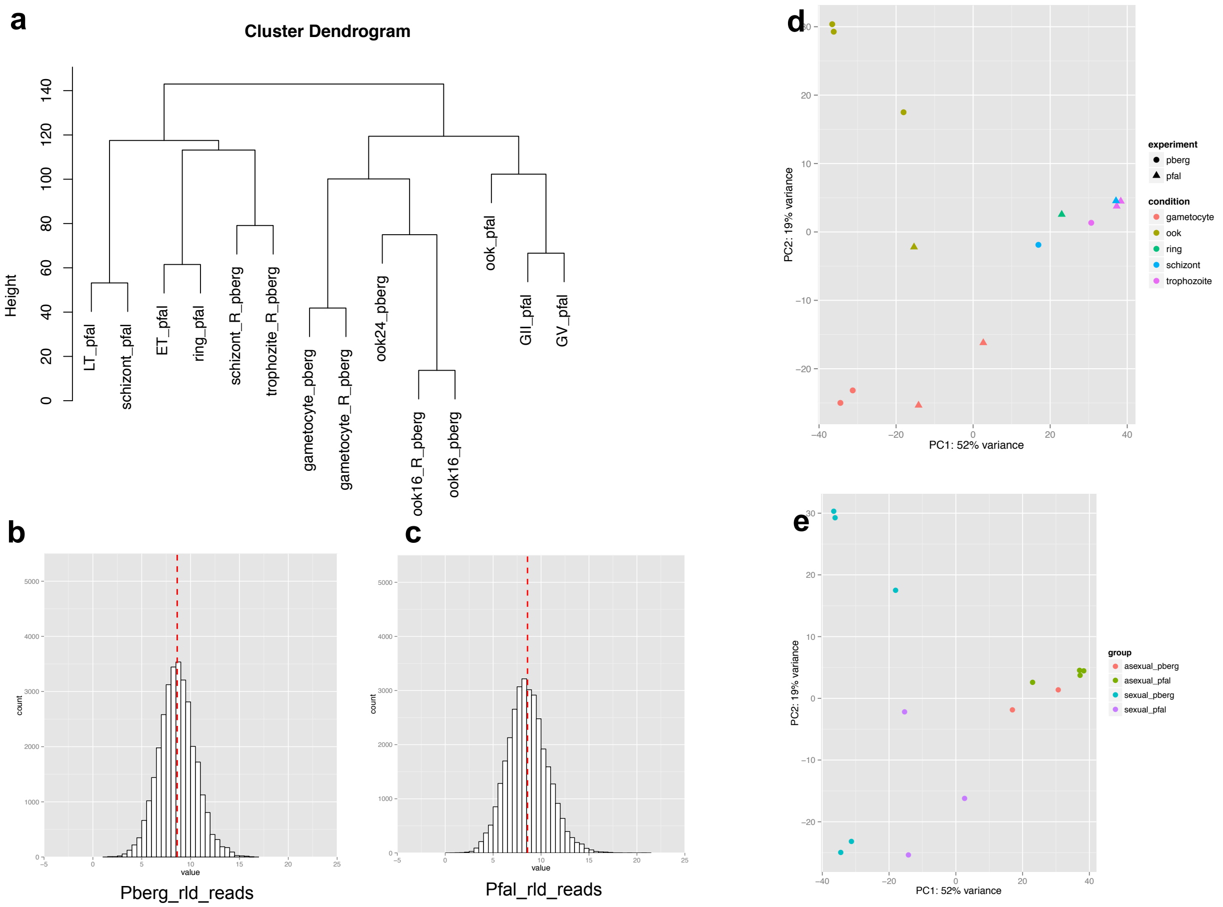

Figure G| Comparison of the transcriptomic data used for constraining the rodent and human infecting species. a) Clustering of *P.berghei* and *P.falciparum* stage specific transcriptomic data using normalized read counts. No batch effect was observed where sexual and asexual stages from each species co-cluster. Distribution of normalized read counts from b) *P. berghei* and c) *P. fal*ciparum stage-specific data. PCA plots based on normalized read counts for d) *P. berghei* and e) *P. falciparum* stage-specific data.

Since it was not clearly specified which gametocyte stage was used for expression profiling of *P. berghei*[66], we have generated two gametocyte models using the same expression data but differing in the objective function to be maximized, namely the objective function of the early gametocyte model was to maximize biomass (growth rate) while that of the late gametocyte stage was to maximize ATP production. Although, this is less accurate in comparison to the *P. falciparum* models for which expression data was available for the early and late gametocyte,

we found high overlap between the predictions made by the stage-specific models from the two species.

High resolution figure legends

S1 Fig. (corresponding Fig. 4 of main text). Stage-specific central metabolic flux patterns in malaria. (a) Correlated reaction sets for iAM-Pf480 were used to define stage and model specific pathways, which were analyzed and compared across different stages. Modularity indices (see methods) were 0.023, 0.024, 0.026, 0.145, 0.022 for the T, Schizont, GII, GV, and Ook stages, respectively. In proliferative versus non-proliferative stages of malaria, there were changes in the patterns of central carbon metabolism, notably the non-oxidative PPP and glycolysis. (b) The direction of flux in the non-oxidative branch of PPP goes towards production of glycolytic intermediates in the Trophozoite, Schizont, GII, and GV stages but not the Ookinete stage. Reversal of non-oxidative pentose phosphate pathway fluxes in the Ook enables provision of ribose 5 phosphate (r5p) needed for the synthesis of nucleotide precursors of DNA. The non-oxidative branch in the schizont is colored in red indicating its coupling to growth rate in this stage (Table G in S1 Tables). Both the oxidative and non-oxidative PPP branches were correlated in GII. Glycolysis was split into upper and lower branches in all stages except GV where the non-oxidative PPP branch was correlated with inositol metabolism. Arrows are omitted from the schizont pathway map to account for reduced flux values relative the other 4 stages. (c) Predicted sampled flux distribution are shown in the non-oxidative branch of PPP (Transketolase; TKT1) and inositol metabolism (myo-inositol-3-phosphate lyase; MI3PS) across all the stages showing increased involvement of inositol metabolism in the GV.

S2 Fig. (corresponding Fig. 5 of main text). Stage-specific essentiality predictions of experimentally validated druggable targets and single-gene deletion experiments. Comprehensive map of experimentally tested treatment targets for P. falciparum with stage-specific model predictions projected (in color) projected on top of the map. Colored reaction pathways correspond to drug inhibition studies (Table C in S1 Tables) and colored reaction names in rectangles correspond to single gene deletion experiments (Table B in S1 Tables). The color legend inset corresponds to iAM-Pf480 predictions. Validated drug targets that are also predictive to reduce growth in proliferative as well as late gametocyte stages are of particular interest. This comprehensive assessment of the model and experimental results enables stratification of existing drugs, new drugs to target, as well as new areas of metabolism warranting further investigation.

S3 Fig. (corresponding Fig. 6 of main text). Species-specific models provide mechanistic explanation for differences in drug response between human- and rodent-infecting malaria species. (a) The core and pan metabolic content of 5 malaria species was identified based on the respective species-specific reconstructions. The core content, illustrated by the intersection of the Venn diagram, is shared by all species. The pan content represents the union of the content across all of the multi-species reconstructions. (b) 14 metabolic reactions differed in their presence across the 5 reconstructed Plasmodium species. (c) Thiamine

pyrophosphokinase (TPK) and (d) Choline kinase (CK) were predicted by the models to be essential for the growth of the rodent-infecting species (P. berghei) while their deletion had no effect on the growth of human and non-human primate species. Differential essentiality of TPK is due to absence of phosphomethylpyrimidine kinase and thiamine-phosphate pyrophosphorylase the rodent-infecting species. In the case of CK, the differential essentiality is due to the absence of phosphoethanolamine N-methyltransferase. (See Table A in S1 Tables for reactions abbreviations and gene-protein-reaction associations). (e) Pantothenate metabolism showed differences in essentiality between stage- and species-specific models. Tables indicate percentage in growth reduction compared to the WT upon deletion of the respective gene. 'X' indicates absence of a reaction from the respective reconstruction, '—' indicates no effect on growth upon deletion of the corresponding reaction and '%' indicates the growth reduction percentage resulting from deletion of the corresponding gene. T: trophozoite, GII: early gametocyte stage, GV: late gametocyte stage, Ook: ookinete.

References

1 Chavali, A. K., Whittemore, J. D., Eddy, J. A., Williams, K. T. & Papin, J. A. Systems analysis of metabolism in the pathogenic trypanosomatid Leishmania major. *Mol Syst Biol* **4**, 177, doi:10.1038/msb.2008.15 (2008).

2 Hsiao, L. L., Howard, R. J., Aikawa, M. & Taraschi, T. F. Modification of host cell membrane lipid composition by the intra-erythrocytic human malaria parasite Plasmodium falciparum. *Biochem J* **274 (Pt 1)**, 121-132 (1991).

3 Jamshidi, N. & Palsson, B. O. Investigating the metabolic capabilities of Mycobacterium tuberculosis H37Rv using the in silico strain iNJ661 and proposing alternative drug targets. *BMC Syst Biol* **1**, 26, doi:10.1186/1752-0509-1-26 (2007).

4 Bender, A., van Dooren, G. G., Ralph, S. A., McFadden, G. I. & Schneider, G. Properties and prediction of mitochondrial transit peptides from Plasmodium falciparum. *Mol Biochem Parasitol* **132**, 59-66 (2003).

5 Reed, J. L., Vo, T. D., Schilling, C. H. & Palsson, B. O. An expanded genome-scale model of Escherichia coli K-12 (iJR904 GSM/GPR). *Genome Biol* **4**, R54, doi:10.1186/gb-2003-4-9-r54 (2003).

6 Thiele, I. & Palsson, B. O. A protocol for generating a high-quality genome-scale metabolic reconstruction. *Nat Protoc* **5**, 93-121, doi:10.1038/nprot.2009.203 (2010).

7 Green, M. L. & Karp, P. D. A Bayesian method for identifying missing enzymes in predicted metabolic pathway databases. *BMC Bioinformatics* **5**, 76, doi:10.1186/1471-2105-5-76 (2004).

8 Muller, I. B., Hyde, J. E. & Wrenger, C. Vitamin B metabolism in Plasmodium falciparum as a source of drug targets. *Trends Parasitol* **26**, 35-43, doi:10.1016/j.pt.2009.10.006 (2010).

9 Cobbold, S. A. *et al.* Kinetic flux profiling elucidates two independent acetyl-CoA biosynthetic pathways in Plasmodium falciparum. *J Biol Chem* **288**, 36338-36350, doi:10.1074/jbc.M113.503557 (2013).

10 Plata, G., Hsiao, T. L., Olszewski, K. L., Llinas, M. & Vitkup, D. Reconstruction and flux-balance analysis of the Plasmodium falciparum metabolic network. *Mol Syst Biol* **6**, 408, doi:10.1038/msb.2010.60 (2010).

11 Niemand, J., Louw, A. I., Birkholtz, L. & Kirk, K. Polyamine uptake by the intraerythrocytic malaria parasite, Plasmodium falciparum. *Int J Parasitol* **42**, 921-929, doi:10.1016/j.ijpara.2012.07.005 (2012).
12 Olszewski, K. L. *et al.* Host-parasite interactions revealed by Plasmodium falciparum metabolomics. *Cell Host Microbe* **5**, 191-199, doi:10.1016/j.chom.2009.01.004 (2009).
13 Lauer, S. *et al.* Vacuolar uptake of host components, and a role for cholesterol and sphingomyelin in malarial infection. *EMBO J* **19**, 3556-3564, doi:10.1093/emboj/19.14.3556 (2000).
14 Miao, J. *et al.* Plasmodium falciparum: generation of pure gametocyte culture by heparin treatment. *Exp Parasitol* **135**, 541-545, doi:10.1016/j.exppara.2013.09.010 (2013).
15 Nam, H. *et al.* Network context and selection in the evolution to enzyme specificity. *Science* **337**, 1101-1104, doi:10.1126/science.1216861 (2012).
16 Jensen, M. D., Conley, M. & Helstowski, L. D. Culture of Plasmodium falciparum: the role of pH, glucose, and lactate. *J Parasitol* **69**, 1060-1067 (1983).
17 Quashie, N. B., Ranford-Cartwright, L. C. & de Koning, H. P. Uptake of purines in Plasmodium falciparum-infected human erythrocytes is mostly mediated by the human equilibrative nucleoside transporter and the human facilitative nucleobase transporter. *Malar J* **9**, 36, doi:10.1186/1475-2875-9-36 (2010).
18 Schellenberger, J., Lewis, N. E. & Palsson, B. O. Elimination of thermodynamically infeasible loops in steady-state metabolic models. *Biophys J* **100**, 544-553, doi:10.1016/j.bpj.2010.12.3707 (2011).
19 Ginsburg, H. & Abdel-Haleem, A. M. Malaria Parasite Metabolic Pathways (MPMP) Upgraded with Targeted Chemical Compounds. *Trends Parasitol* **32**, 7-9, doi:10.1016/j.pt.2015.10.003 (2016).
20 Monk, J. M. *et al.* Genome-scale metabolic reconstructions of multiple Escherichia coli strains highlight strain-specific adaptations to nutritional environments. *Proc Natl Acad Sci U S A* **110**, 20338-20343, doi:10.1073/pnas.1307797110 (2013).
21 Plata, G., Gottesman, M. E. & Vitkup, D. The rate of the molecular clock and the cost of gratuitous protein synthesis. *Genome Biol* **11**, R98, doi:10.1186/gb-2010-11-9-r98 (2010).
22 Chiappino-Pepe, A., Tymoshenko, S., Ataman, M., Soldati-Favre, D. & Hatzimanikatis, V. Bioenergetics-based modeling of Plasmodium falciparum metabolism reveals its essential genes, nutritional requirements, and thermodynamic bottlenecks. *PLoS Comput Biol* **13**, e1005397, doi:10.1371/journal.pcbi.1005397 (2017).
23 Carey, M. A., Papin, J. A. & Guler, J. L. Novel Plasmodium falciparum metabolic network reconstruction identifies shifts associated with clinical antimalarial resistance. *BMC Genomics* **18**, 543, doi:10.1186/s12864-017-3905-1 (2017).
24 Trapnell, C. *et al.* Differential gene and transcript expression analysis of RNA-seq experiments with TopHat and Cufflinks. *Nat Protoc* **7**, 562-578, doi:10.1038/nprot.2012.016 (2012).
25 Becker, S. A. & Palsson, B. O. Context-specific metabolic networks are consistent with experiments. *PLoS Comput Biol* **4**, e1000082, doi:10.1371/journal.pcbi.1000082 (2008).
26 Love, M. I., Huber, W. & Anders, S. Moderated estimation of fold change and dispersion for RNA-seq data with DESeq2. *Genome Biol* **15**, 550, doi:10.1186/s13059-014-0550-8 (2014).
27 Anders, S., Pyl, P. T. & Huber, W. HTSeq--a Python framework to work with high-throughput sequencing data. *Bioinformatics* **31**, 166-169, doi:10.1093/bioinformatics/btu638 (2015).
28 Sing, T., Sander, O., Beerenwinkel, N. & Lengauer, T. ROCR: visualizing classifier performance in R. *Bioinformatics* **21**, 3940-3941, doi:10.1093/bioinformatics/bti623 (2005).

29 MacRae, J. I. *et al.* Mitochondrial metabolism of sexual and asexual blood stages of the malaria parasite Plasmodium falciparum. *BMC Biol* **11**, 67, doi:10.1186/1741-7007-11-67 (2013).

30 Madrid, D. C., Ting, L. M., Waller, K. L., Schramm, V. L. & Kim, K. Plasmodium falciparum purine nucleoside phosphorylase is critical for viability of malaria parasites. *J Biol Chem* **283**, 35899-35907, doi:10.1074/jbc.M807218200 (2008).

31 Plouffe, D. M. *et al.* High-Throughput Assay and Discovery of Small Molecules that Interrupt Malaria Transmission. *Cell Host Microbe* **19**, 114-126, doi:10.1016/j.chom.2015.12.001 (2016).

32 Lopez-Barragan, M. J. *et al.* Directional gene expression and antisense transcripts in sexual and asexual stages of Plasmodium falciparum. *BMC Genomics* **12**, 587, doi:10.1186/1471-2164-12-587 (2011).

33 Leinonen, R., Sugawara, H., Shumway, M. & International Nucleotide Sequence Database, C. The sequence read archive. *Nucleic Acids Res* **39**, D19-21, doi:10.1093/nar/gkq1019 (2011).

34 Kim, D. *et al.* TopHat2: accurate alignment of transcriptomes in the presence of insertions, deletions and gene fusions. *Genome Biol* **14**, R36, doi:10.1186/gb-2013-14-4-r36 (2013).

35 Li, H. *et al.* The Sequence Alignment/Map format and SAMtools. *Bioinformatics* **25**, 2078-2079, doi:10.1093/bioinformatics/btp352 (2009).

36 Machado, D. & Herrgard, M. Systematic evaluation of methods for integration of transcriptomic data into constraint-based models of metabolism. *PLoS Comput Biol* **10**, e1003580, doi:10.1371/journal.pcbi.1003580 (2014).

37 Kusuda, H. *et al.* Ectopic expression of myo-inositol 3-phosphate synthase induces a wide range of metabolic changes and confers salt tolerance in rice. *Plant Sci* **232**, 49-56, doi:10.1016/j.plantsci.2014.12.009 (2015).

38 Ke, H. *et al.* Genetic investigation of tricarboxylic acid metabolism during the Plasmodium falciparum life cycle. *Cell Rep* **11**, 164-174, doi:10.1016/j.celrep.2015.03.011 (2015).

39 Josling, G. A. & Llinas, M. Sexual development in Plasmodium parasites: knowing when it's time to commit. *Nat Rev Microbiol* **13**, 573-587, doi:10.1038/nrmicro3519 (2015).

40 McNamara, C. W. *et al.* Targeting Plasmodium PI(4)K to eliminate malaria. *Nature* **504**, 248-253, doi:10.1038/nature12782 (2013).

41 Macrae, J. I. *et al.* Plasmodium falciparum is dependent on de novo myo-inositol biosynthesis for assembly of GPI glycolipids and infectivity. *Mol Microbiol* **91**, 762-776, doi:10.1111/mmi.12496 (2014).

42 Cobbold, S. A., Martin, R. E. & Kirk, K. Methionine transport in the malaria parasite Plasmodium falciparum. *Int J Parasitol* **41**, 125-135, doi:10.1016/j.ijpara.2010.09.001 (2011).

43 Sengupta, A. *et al.* Global host metabolic response to Plasmodium vivax infection: a 1H NMR based urinary metabonomic study. *Malar J* **10**, 384, doi:10.1186/1475-2875-10-384 (2011).

44 Bulusu, V., Jayaraman, V. & Balaram, H. Metabolic fate of fumarate, a side product of the purine salvage pathway in the intraerythrocytic stages of Plasmodium falciparum. *J Biol Chem* **286**, 9236-9245, doi:10.1074/jbc.M110.173328 (2011).

45 Yang, J. *et al.* The I-TASSER Suite: protein structure and function prediction. *Nat Methods* **12**, 7-8, doi:10.1038/nmeth.3213 (2015).

46 Roy, A., Kucukural, A. & Zhang, Y. I-TASSER: a unified platform for automated protein structure and function prediction. *Nat Protoc* **5**, 725-738, doi:10.1038/nprot.2010.5 (2010).

47 Zhang, Y. I-TASSER server for protein 3D structure prediction. *BMC Bioinformatics* **9**, 40, doi:10.1186/1471-2105-9-40 (2008).
48 Woo, Y. H. *et al.* Chromerid genomes reveal the evolutionary path from photosynthetic algae to obligate intracellular parasites. *Elife* **4**, e06974, doi:10.7554/eLife.06974 (2015).
49 Sana, T. R. *et al.* Global mass spectrometry based metabolomics profiling of erythrocytes infected with Plasmodium falciparum. *PLoS One* **8**, e60840, doi:10.1371/journal.pone.0060840 (2013).
50 Ghigo, D. *et al.* Erythrocyte stages of Plasmodium falciparum exhibit a high nitric oxide synthase (NOS) activity and release an NOS-inducing soluble factor. *J Exp Med* **182**, 677-688 (1995).
51 Hyde, J. E. *et al.* Plasmodium falciparum: a paradigm for alternative folate biosynthesis in diverse microorganisms? *Trends Parasitol* **24**, 502-508, doi:10.1016/j.pt.2008.08.004 (2008).
52 Percario, S. *et al.* Oxidative stress in malaria. *Int J Mol Sci* **13**, 16346-16372, doi:10.3390/ijms131216346 (2012).
53 Cobbold, S. A., Llinas, M. & Kirk, K. Sequestration and metabolism of host cell arginine by the intraerythrocytic malaria parasite Plasmodium falciparum. *Cell Microbiol*, doi:10.1111/cmi.12552 (2015).
54 Carbary, L. J. A plea to remember the constant need for organ donations. *RN* **54**, 10, 12 (1991).
55 Fry, M. & Beesley, J. E. Mitochondria of mammalian Plasmodium spp. *Parasitology* **102 Pt 1**, 17-26 (1991).
56 Goldberg, D. E., Slater, A. F., Cerami, A. & Henderson, G. B. Hemoglobin degradation in the malaria parasite Plasmodium falciparum: an ordered process in a unique organelle. *Proc Natl Acad Sci U S A* **87**, 2931-2935 (1990).
57 Bandini, G. *et al.* Phosphoglucomutase is absent in Trypanosoma brucei and redundantly substituted by phosphomannomutase and phospho-N-acetylglucosamine mutase. *Mol Microbiol* **85**, 513-534, doi:10.1111/j.1365-2958.2012.08124.x (2012).
58 Cowman, A. F. & Crabb, B. S. Invasion of red blood cells by malaria parasites. *Cell* **124**, 755-766, doi:10.1016/j.cell.2006.02.006 (2006).
59 Sanz, S. *et al.* The disruption of GDP-fucose de novo biosynthesis suggests the presence of a novel fucose-containing glycoconjugate in Plasmodium asexual blood stages. *Sci Rep* **6**, 37230, doi:10.1038/srep37230 (2016).
60 Serricchio, M. & Butikofer, P. An essential bacterial-type cardiolipin synthase mediates cardiolipin formation in a eukaryote. *Proc Natl Acad Sci U S A* **109**, E954-961, doi:10.1073/pnas.1121528109 (2012).
61 Reader, J. *et al.* Nowhere to hide: interrogating different metabolic parameters of Plasmodium falciparum gametocytes in a transmission blocking drug discovery pipeline towards malaria elimination. *Malar J* **14**, 213, doi:10.1186/s12936-015-0718-z (2015).
62 O'Hara, J. K. *et al.* Targeting NAD+ metabolism in the human malaria parasite Plasmodium falciparum. *PLoS One* **9**, e94061, doi:10.1371/journal.pone.0094061 (2014).
63 Botte, C. Y. *et al.* Atypical lipid composition in the purified relict plastid (apicoplast) of malaria parasites. *Proc Natl Acad Sci U S A* **110**, 7506-7511, doi:10.1073/pnas.1301251110 (2013).
64 Dereeper, A. *et al.* Phylogeny.fr: robust phylogenetic analysis for the non-specialist. *Nucleic Acids Res* **36**, W465-469, doi:10.1093/nar/gkn180 (2008).
65 Dereeper, A., Audic, S., Claverie, J. M. & Blanc, G. BLAST-EXPLORER helps you building datasets for phylogenetic analysis. *BMC Evol Biol* **10**, 8, doi:10.1186/1471-2148-10-8 (2010).

66 Otto, T. D. *et al.* A comprehensive evaluation of rodent malaria parasite genomes and gene expression. *BMC Biol* **12**, 86, doi:10.1186/s12915-014-0086-0 (2014).
